# Ozone photochemistry in fresh biomass burning smoke over the United States

**DOI:** 10.1126/sciadv.ads2157

**Published:** 2026-02-06

**Authors:** Lixu Jin, Matthew M. Coggon, Wade Permar, Julieta F. Juncosa Calahorrano, Brett B. Palm, Georgios I. Gkatzelis, Michael A. Robinson, Ilann Bourgeois, Samuel R. Hall, Jeff Peischl, Kirk Ullmann, Joel A. Thornton, Carsten Warneke, Frank Flocke, Emily V. Fischer, Robert J. Yokelson, Lu Hu

**Affiliations:** ^1^ Department of Chemistry and Biochemistry, University of Montana, Missoula, MT, USA.; ^2^ Chemical Sciences Laboratory, National Oceanic and Atmospheric Administration, Boulder, CO, USA.; ^3^ Department of Soil, Water, and Climate, University of Minnesota, St. Paul, MN, USA.; ^4^ Atmospheric Chemistry Observations & Modeling Laboratory, NSF National Center for Atmospheric Research, Boulder, CO, USA.; ^5^ Institute of Climate and Energy Systems, ICE-3: Troposphere, Forschungszentrum Jülich, Jülich 52428, Germany.; ^6^ Cooperative Institute for Research in Environmental Sciences, University of Colorado Boulder, Boulder, CO, USA.; ^7^ Department of Chemistry, University of Colorado Boulder, Boulder, CO, USA.; ^8^ CARRTEL, Université Savoie Mont Blanc, INRAE, Thonon-les-Bains, France.; ^9^ Department of Atmospheric and Climate Science, University of Washington, Seattle, WA, USA.; ^10^ Department of Atmospheric Science, Colorado State University, Fort Collins, CO, USA.

## Abstract

The first 5 hours of aging in biomass burning plumes can strongly affect ozone photochemistry. We examine how volatile organic compounds (VOCs), nitrogen oxides, and nitrous acid influence hydroxyl radical, ozone, and peroxyacetyl nitrate (PAN) based on three aircraft campaigns over the United States. Our analyses reveal variable, highly elevated hydroxyl radical concentrations in the first 2 hours, resulting in evident fire-to-fire variability in VOCs oxidation and in ozone and PAN production. About 40 to 70% of the variability is explained by chemical aging. Ozone production in the plumes is usually VOC-limited for the first 2 hours and then nitrogen oxide limited downwind. Box model results for hydroxyl radical, ozone, and most VOCs, using the full, explicit Master Chemical Mechanism (MCM) mechanism, suggest no major gaps in the current best knowledge of gas-phase chemistry. However, the MCM sometimes overestimates PAN due to underestimated nitrogen oxide sinks. GEOS-Chem, a widely used chemical transport model with a reduced mechanism, generally underperforms because of incomplete VOC representation. We identify these critical pathways to guide future model development.

## Teaser

Identifying key knowledge gaps and outlining steps to improve ozone photochemistry modeling in fresh biomass-burning plumes.

## INTRODUCTION

Predicting ozone in biomass burning (BB)–influenced environments is challenging, especially for chemical transport models (CTMs) (*1*). Errors, in modeling urban, rural, and remote areas affected by BB (*2*–*4*), lead to difficulties in protecting public health and estimating ozone radiative forcing (*5*). Recent progress has been made in improving the understanding of BB emissions, chemistry, plume injections, and transport (*6*–*9*). This progress is partly due to the availability of fire-targeting laboratory and field observations (*10*–*12*), mechanism development (*13*, *14*), and improvements in remote sensing techniques (*15*, *16*). A few recent studies have investigated and modeled the chemical evolution in highly polluted BB plumes at the plume level (*8*, *17*–*19*). However, how to transfer these findings from the plume level to the regional or global scales remains unclear. Here, as a first step toward bridging this gap, we assess current knowledge of photochemistry involving hydroxyl radical (OH), volatile organic compounds (VOCs), ozone, and reactive nitrates in highly polluted BB plumes and identify key underrepresented chemical processes that limit accurate modeling in BB environments.

Despite recent advances, modeling photochemistry in BB plumes remains highly uncertain, largely due to incomplete representation of VOCs. CTMs typically use simplified chemical mechanisms with reduced numbers of VOC species and reactions due to computational limitations. For example, CTM mechanisms such as Statewide Air Pollution Research Center version 11 (SAPRC-11), Model for OZone and Related chemical Tracers tropospheric mechanism T1 (MOZART-T1), and GEOS-Chem simulate 150 to 300 species and 350 to 800 reactions, including 20 to 40 VOCs, many of which are represented as lumped species. By comparison, the near-explicit Master Chemical Mechanism (MCM) is at least 20 times more complex (6000 species, 17,000 reactions, 142 nonmethane VOCs) (*20*–*27*). These chemical mechanisms are often developed and validated for urban, forest, or remote tropospheric environments. Recent studies have suggested that the CTM mechanisms cannot fully represent BB plumes, which have a much more complex chemical composition than traditionally studied urban environments. Specifically, CTMs are estimated to miss at least half of the initial OH reactivity from VOCs (OHR_VOC_) in BB emissions due to incomplete representation of VOC species (*9*, *17*). The omitted VOCs also partly explain the missing sources of secondary oxygenated VOCs (OVOCs) and ozone that are not captured by CTMs in BB smoke (*2*, *6*). Furthermore, their chemical evolution in fresh BB plumes has yet to be thoroughly assessed within CTM mechanisms, partly because chemical processes are intertwined with other BB processes and thus are difficult to evaluate in CTM platforms.

Even the near-explicit MCM does not guarantee accurate ozone predictions. Recent ground-based investigations show that MCM systematically overestimates ozone production by overestimating peroxy radicals (XO_2_) when smoke ages beyond 12 hours (*28*). Conversely, in plumes aged less than 5 hours, MCM has been found to underestimate ozone abundance due to the absence of XO_2_ from key VOC precursors such as furanoids (*14*, *28*). Limited evidence suggests that these missing XO_2_ sources in the fresh smoke could result in underestimating the nitrogen oxides’ (NO*_x_* = NO + NO_2_) loss rate and/or overestimating the formation of peroxyacetyl nitrate (PAN) in MCM (*29*). Either error would compromise the model’s ability to predict downwind ozone. Furthermore, in studies using MCM, the number of VOCs for model initialization varies widely, ranging from 15 to 150 species, which likely contributes to the different conclusions (*30*–*32*). Therefore, a systematic comparison of different chemical mechanisms and VOC representations is necessary to assess the current understanding of photochemistry in BB smoke.

In this study, we examine the current state-of-the-art gas-phase photochemistry in near-field BB plumes based on three aircraft campaigns over the United States, as increasing evidence suggests that the first few hours of chemical evolution can strongly affect downwind ozone photochemistry (*1*, *33*–*35*). We assess the current knowledge of photochemistry in highly polluted BB plumes by comparing recent aircraft observations to both a near-explicit chemical mechanism that represents our best knowledge of atmospheric chemistry and a reduced chemical mechanism commonly used in global models. We evaluate the chemical mechanisms within a box modeling platform, thus effectively isolating other BB processes and identifying key pathways needed to improve the modeling of BB plume chemistry.

## RESULTS

### No major gaps in explicit gas-phase chemistry when predicting OH

We calculate OH concentrations at the center of BB plumes using changing ratios of two trace gases that are coemitted from the same emission source and degrade at different rates against OH (*36*, *37*). In Eq. 1, [OH]_cal_ is the calculated average OH concentrations between two subsequent transects sampled at t(i) and t(i+1) [t is plume age of the sampled air mass; Δt=t(i+1)−t(i)]. To estimate [OH]_cal_ between the fire source t(0) and the first transect t(1) sampled by aircraft, we use [VOC:CO] at t(0) from a recent compilation of emission ratios for temperate forests (*38*)*.* The *k*_OH+VOC_ and *k*_OH+CO_ are the reaction rates of VOCs and CO with OH.

Sampling subsequent transects capture short-interval OH evolution that would be masked by the traditional method using wider separated transects [e.g., t(0) and t(i)]. Moreover, because we calculate [OH]_cal_ from subsequent plume transects sampled by the aircraft (<~20 min apart), BB plumes were typically emitted at similar times and under similar fire conditions. Thus, our [OH]_cal_ depends less on assuming Lagrangian sampling. Even if one transect pair is affected by changing emissions, the erroneous [OH]_cal_ would not propagate across the overall analysis.

Overall, potential biases in this approach can arise from the following: (i) estimating plume physical age, (ii) non-OH chemical sinks or production affecting the selected VOC and CO, (iii) uncertainties in the VOC concentrations or their reaction rates with OH, (iv) changing emissions between two subsequent transects in the pseudo-Lagrangian sampling, and (v) diluted smoke from other nearby fires

$$\begin{array}{cc} {{\left[\text{OH}\right]}_{\text{cal}}|}_{t\left(i\right)}= & \frac{1}{\Delta t\left(k_{\text{OH}+\text{VOC}}-k_{\text{OH}+\text{CO}}\right)}\\ & \times \left\{\text{ln}\left[{\left.\frac{\left[\text{VOC}\right]}{\left[\text{CO}\right]}\right|}_{t\left(i\right)}\right]-\text{ln}\left[{\left.\frac{\left[\text{VOC}\right]}{\left[\text{CO}\right]}\right|}_{t\left(i+1\right)}\right]\right\} \end{array}$$ 1

To minimize potential errors in particular [VOC:CO] pairs, we select VOCs that are primarily consumed by OH oxidation (>90% of overall sinks per MCM), with little to no secondary production, and lifetimes of 4 to 8 hours (at OH = 1 × 10^6^ molecules cm^−3^). The strict criteria reduce the initial pool of ~90 VOCs to three compounds: furan, furfural, and furanone. Loosening the lifetime criteria adds a few more VOCs but does not affect the overall conclusions. We also compare the OH reaction rate constants (*k*_OH+VOC_) used in the mechanisms with recent measurements and assessments of *k*_OH+VOC_ and find that uncertainties in *k*_OH+VOC_ do not affect our findings (*39*).

We then assess the robustness and uncertainty in [OH]_cal_ in three ways using data from three BB plumes where all three VOCs were measured (fig. S1). First, using observations, we find that [OH]_cal_ across the three VOC-CO pairs shows good agreement, within 30% of their mean, during the first 2.5 hours of physical aging, confirming the consistency of the selected VOCs. However, the coefficient of variance (CV) for [OH]_cal_ increases with time, likely due to higher measurement requirements for detecting smaller VOC decays, nonideal sampling of the plume center, or the mixing of other sources in more aged and diluted plumes (i.e., CV = 50% when plume ages exceed 2.5 hours).

Second, we use modeled VOC and CO as pseudo-observations, simulating ideal Lagrangian sampling of the plume center to validate the mathematical soundness of the calculation method (Eq. 1) in an environment where the “true” OH is known. We calculate [OH]_cal_ using modeled VOC/CO and compare that with OH concentrations directly outputted from the model ([OH]_model_). Only the MCM initialized with explicit VOCs (MCM_BBVOC_, see Materials and Methods) includes all three selected VOCs; therefore, this OH evaluation is conducted using MCM_BBVOC_. The [OH]_cal_ from the three different modeled VOC-CO pairs agrees with each other within ~10% and agrees with [OH]_model_ within ~20% during the first 5 hours of physical aging across five BB plumes. These results suggest that, in an idealized sampling scenario, the approach of estimating OH proves to be reliable, at least within the time frame in this work.

Third, when comparing model results with observations, [OH]_model_ from MCM_BBVOC_ agrees with the average [OH]_cal_ based on measurements within 40% in three selected BB plumes. The agreement is especially good for the Taylor Creek Fire (<10%), while the [OH]_model_ underestimates [OH]_cal_ by ~30 to 40% in the other two BB plumes. As discussed, OH is sensitive to various photolysis reactions and, thus, to the location and aerosol compositions and/or concentrations within the plume transects. OH concentrations at plume edges can be up to an order of magnitude higher than those in the plume center, largely due to higher *J* values associated with lower aerosol concentrations (*8*, *19*, *40*). Therefore, failing to sample the center of large plumes could artificially inflate calculated OH concentrations, which may partly explain the negative model bias.

Despite these challenges, MCM_BBVOC_ reproduces the observation-constrained OH concentrations within 10 to 40% in three selected BB plumes where measurements of all three VOCs are available (fig. S1). As detailed in the following section, we conduct an OH budget closure analysis across five selected BB plumes, which shows that MCM_BBVOC_ accurately reproduces both the sources and sinks of plume-center OH within 20%. These results suggest that there are no major knowledge gaps in the current best understanding of gas-phase chemistry when predicting OH concentrations. Recently, Xu *et al.* (*8*) concluded that there are no major gaps in ozone chemistry in fresh BB plumes, and our analysis confirms this for OH chemistry.

### Within-plume OH abundance, production, and sinks

**Fig. 1.**
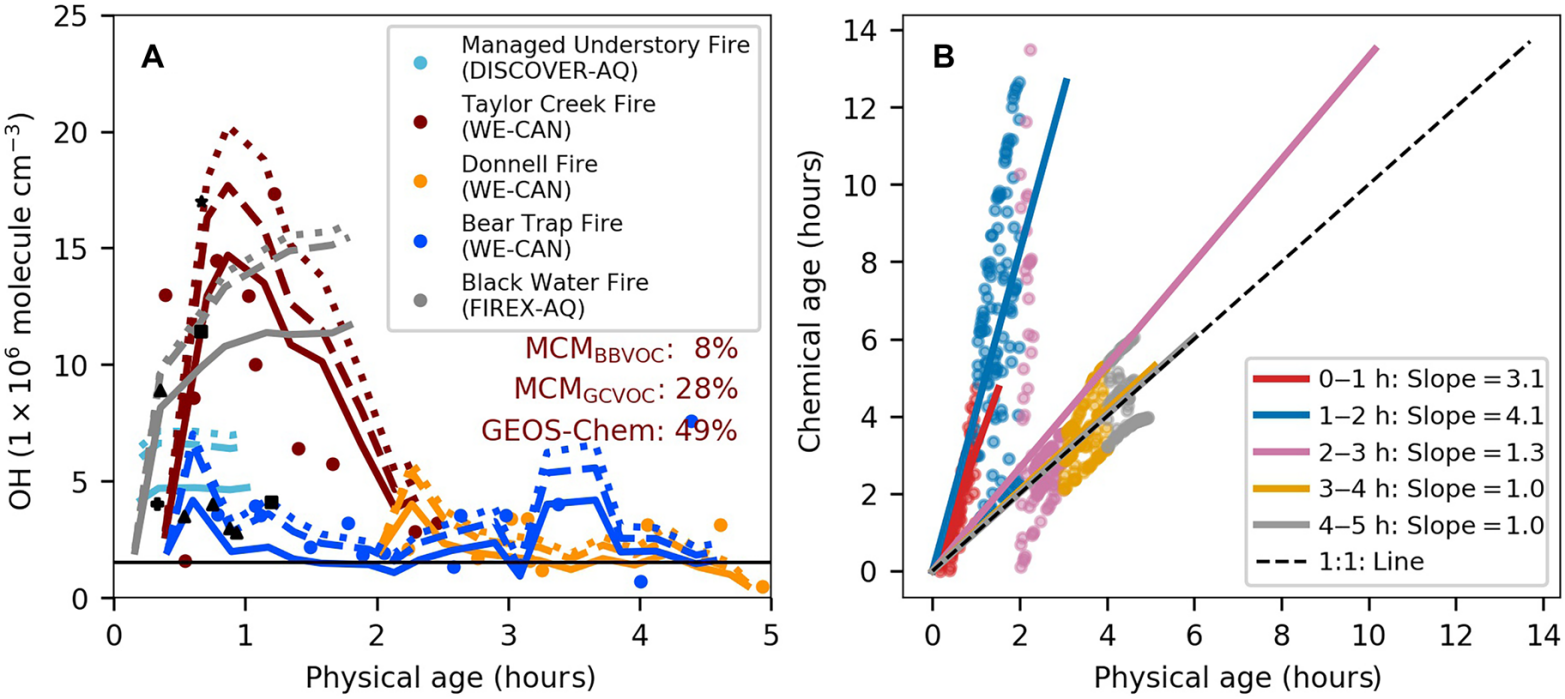
Plume-center OH evolution and chemical-physical age relationships in BB plumes. (**A**) Evolution of plume-center OH concentrations in five BB plumes. OH concentrations calculated using three measured VOCs and CO are shown as circles, colored by different BB plumes. Also shown are the corresponding modeled OH concentrations from MCM_BBVOC_ (solid lines), MCM_GCVOC_ (dashed lines), and GEOS-Chem (dotted lines). The thick black horizontal line represents the assumed ambient OH level (1.5 × 10^6^ molecules cm^−3^). Normalized mean biases (NMBs) between models and observations for the Taylor Creek Fire (red text) are shown as inset. The NMBs are –21% for MCM_BBVOC_, 4% for MCM_GCVOC_, and 17% for GEOS-Chem in Donnell Fire and are –41% for MCM_BBVOC_, –17% for MCM_GCVOC_, and –5% for GEOS-Chem in Bear Trap Fire. Additional black solid symbols are OH concentrations from previous studies, including Hobbs *et al.* (*41*) (star; savanna fire), Yokelson *et al.* (*43*) (square; agricultural and/or deforestation fire), Palm *et al.* (*40*) (filled cross; temperate forest fires), and Akherati *et al.* (*42*) (triangles; temperate forest fires). (**B**) Relationship between chemical and physical age in the five BB plumes using MCM_BBVOC_. Results are binned every physical minute (dots). Also shown are linear regression lines between chemical and physical plume age. Both dots and lines are colored by distinct hours of physical aging. The 1:1 line is shown as the dashed black line. h, hours.

Figure 1A summarizes the evolution of OH concentrations in BB plumes within the first 5 hours of physical aging from this study and several other studies. We show notable variability in OH concentrations across different BB plumes, but average [OH]_cal_ is consistently elevated in young smoke compared to typical ambient OH concentrations (~1.5 × 10^6^ molecules cm^−3^). For example, two large fires, including Taylor Creek and Black Water fires, show rapid increases in OH concentrations during the first 1 to 2 hours after emission, reaching ~10 to 15 × 10^6^ molecules cm^−3^ (averaged using [OH]_model_; >10 times higher than the assumed ambient levels), attributed to their relatively high HONO concentration (table S3). The small fire sampled during DISCOVER-AQ (Deriving Information on Surface Conditions from Column and Vertically Resolved Observations Relevant to Air Quality) did not show such high OH concentrations, partly due to the relatively short sampling duration; this plume was sampled for <1 hour of plume aging. However, elevated [OH] levels of ~5 × 10^6^ molecules cm^−3^ are present. Across the BB plumes, after the initial 1 to 2 hours, OH concentrations decrease at different rates toward ambient levels over the timescale of hours, with several outlier values caused by sampling uncertainties in older, more diluted smoke.

Previous observations of OH in BB plumes have primarily involved calculating average OH concentrations between the fire source and the first transects measured by aircraft using Eq. 1 (*18*, *40*–*42*), with only one direct OH measurement reported so far (*43*). Estimated with different trace gas pairs, five BB plumes in the western US during WE-CAN (Western wildfire Experiment for Cloud chemistry, Aerosol absorption and Nitrogen) showed [OH]_cal_ of 3 to 10 × 10^6^ molecules cm^−3^ over the first 21 to 56 min (*40*, *42*). Other reported [OH]_cal_ in BB includes 5 (±1) × 10^6^ molecules cm^−3^ averaged over the first 5 hours in a California chaparral fire and ~17 × 10^6^ molecules cm^−3^ averaged over the first 45 min in a South African savanna fire (*41*, *44*). On the basis of direct measurements in a small Mexican BB plume, OH concentrations were 11.4 × 10^6^ molecules cm^−3^ in smoke aged 22 to 43 min and 4.1 × 10^6^ molecules cm^−3^ in smoke aged 44 to 65 min (*43*). These previous studies, alongside our current work, consistently show elevated OH concentrations within the first 1 to 2 hours of BB plume aging.

To the best of our knowledge, our analysis is the first to track the temporal evolution of OH with reasonable precision across the first 5 hours of aging at BB plume centers. The photochemical oxidation within these young BB plumes is notably faster than under ambient conditions. This is primarily driven by elevated OH concentrations, which shorten the lifetime of reactive compounds and accelerate the production of secondary species. The amount of photochemical oxidation can be quantified using the concept of “chemical age,” which is defined as the equivalent time required at ambient OH concentrations to achieve the same integrated OH exposure (Δt × [OH]). Here, we use [OH]_model_ from MCM_BBVOC_ to calculate the chemical age, as the model accurately reproduces [OH]_cal_ and provides high temporal resolution across different BB plumes. In the first 2 hours of physical aging (based on travel time from the source), the modeled chemical age of the BB plumes studied in this work is ~3 to 4 times their physical age; thus, 1 hour of physical aging in these fresh plumes is equivalent to 3 to 4 hours of photochemical reactions under typical ambient environments (Fig. 1B). This finding illustrates that standard “time since emission” clock could mask the true extent of chemical reactions in fresh BB smoke.

We show that chemical age explains the variability in the oxidation of total VOCs, ozone, and PAN across different BB plumes. Converting physical to chemical age can reduce the variability of oxidation rates across fires from a factor of 4 to a factor of 2 for these compounds. The result indicates that differences in photochemical activity are key to understanding chemical evolution across BB plumes. Specifically, chemical age explains around 40% of the variability in total VOC decay in fresh smoke, as indicated by the evolution of OHR_VOC_ (fig. S2). However, individual VOC species may not be consistently resolved by chemical age (e.g., furanoids and maleic anhydride; fig. S2), suggesting that additional influences must be considered to fully understand plume-to-plume differences. A similar level of accountability is found for the production of ozone and PAN, where chemical age explains 50 to 70% of the variability across BB plumes (see the following sections on ozone and PAN for more details). Xu *et al.* (*8*) showed that OH exposure (or chemical age) is a useful predictor for ozone production. Our work extends this concept further by showing that chemical age also captures the oxidation of other key compounds.

**Fig. 2.**
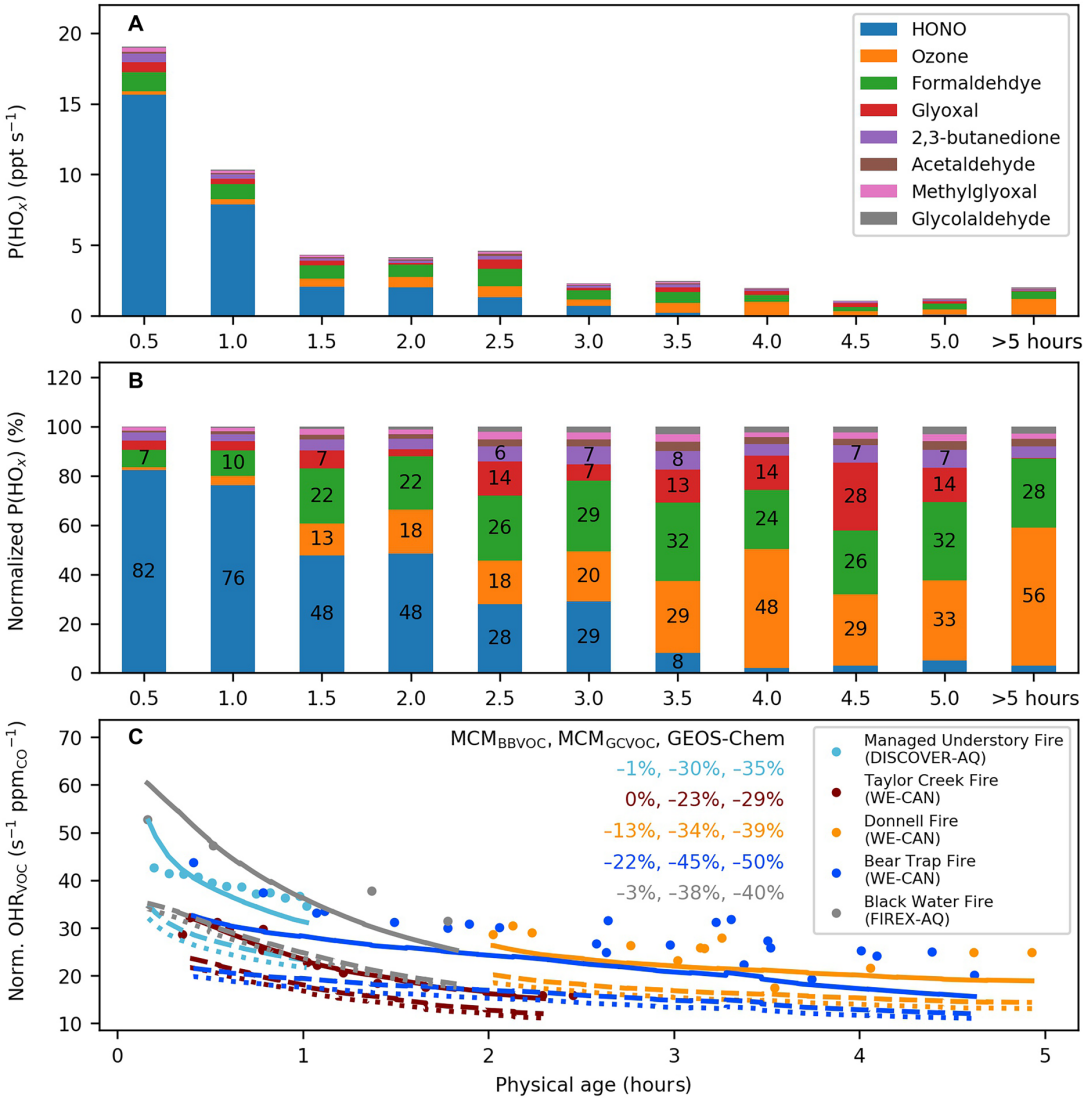
OH budget closure analysis across five selected BB plumes. (**A**) Average HO*_x_* production rates [P(HO*_x_*), HO*_x_* = OH + HO_2_] of different radical sources versus physical age across five BB plumes based on observations. (**B**) Average relative contribution of different radical sources to P(HO*_x_*) versus plume age based on observations. (**C**) Normalized VOC OH reactivity (OHR_VOC_) versus plume age, with observations shown as dots and models as lines (MCM_BBVOC_ as solid lines; MCM_GCVOC_ as dashed lines; and GEOS-Chem as dotted lines). The individual HO*_x_* precursors are colored in (A) and (B) and are averaged across five BB plumes. The different BB plumes are colored in (C), with the inset showing the NMBs between models and observations. Norm., Normalized.

In addition, chemical evolution in young and large wildfire plumes is even faster at the plume edges compared to the plume centers modeled in this work (*40*). This is evidenced by higher ratios of excess ozone, PAN, or maleic anhydride to excess CO at the plume edges (*19*), indicating higher net photochemical production at the edges. As BB plumes age, the incremental (“instantaneous”) ratios of chemical-to-physical age decrease, transitioning from 4 to 1 between the second hour of physical aging and the more aged smoke, reflecting a shift toward ambient atmospheric conditions. This decreasing trend is attributed to the rapid HONO depletion and atmospheric dilution (Fig. 2A).

The rapid rise and fall of OH concentrations reflect dynamic changes in the sources and sinks of OH within fresh BB plumes. We address these changes through a budget closure analysis for both observations and model simulations. For OH sources, we use total radical production [P(HO*_x_*), where HO*_x_* = OH + HO_2_] as a proxy because of the rapid interconversion between OH and HO_2_ (see section S1 for detailed methodologies) (*45*). For OH sinks, we focus on VOC OH reactivity {${\text{OHR}}_{\text{VOC}}=\sum k_{\text{OH}+\text{VOC}\left(i\right)}\times \left[\text{VOC}\left(i\right)\right]$}, which accounts for ~80% of total OH loss (*17*). We note that OH loss caused by aerosol uptakes is negligible (<5%) (*46*, *47*), thus exclusion of aerosol uptakes does not influence our gas-phase OH analysis.

Figure 2 illustrates the evolution of P(HO*_x_*) from observations and OHR_VOC_ based on both observational and model results. In the first hour of physical aging, HONO photolysis is the dominant radical source, accounting for ~(75 ± 22)% of observed P(HO*_x_*) on average across the BB plumes, corroborating recent findings (*8*, *43*, *45*, *48*). HONO continues to contribute ~30% to the observed P(HO*_x_*) in plumes aged 2 to 3 hours. The important role of HONO in slightly aged smoke likely reflects its secondary production from NO_2_ reactive uptake and particulate nitrate (pNO_3_) photolysis, but it is also subject to uncertainties as HONO measurements approach their detection limit (~10 parts per trillion by volume) (*45*, *49*). We note that the first–2-hour P(HO*_x_*) budget is influenced by the Taylor Creek Fire, which exhibited relatively high HONO concentrations, but this case represents one of the few available datasets with direct, high-quality HONO measurements and therefore provides one of the most robust observational constraint on early-plume radical chemistry to date.

As plumes further age from the second to the fifth hour, OVOC photolysis becomes an important radical source, accounting for 40 to 60% of observed P(HO*_x_*). Key contributors among OVOCs include formaldehyde (27 ± 4%; mean ± SD), glyoxal (11 ± 8%), diacetyl (also called 2,3-butanedione or biacetyl; 6 ± 1%), acetaldehyde (3 ± 1%), methylglyoxal (3 ± 0%), and glycolaldehyde (2 ± 1%). Specifically, the budget analysis confirms that diacetyl, which is rarely measured and modeled in BB, could be a substantial contributor to PAN formation (*50*, *51*). Furthermore, as the plumes age and dilute into the background air, the importance of ozone photolysis increases, ultimately accounting for half of observed P(HO*_x_*) in plumes older than 5 hours.

We then compare key results from three simulations with observations and against each other using different chemical mechanisms or numbers of initialized VOCs. The simulations include MCM_BBVOC_ (MCM + explicit VOC), MCM_GCVOC_ (MCM + CTM VOCs), and standard GEOS-Chem, as detailed in Materials and Methods. Our analysis reveals that MCM_BBVOC_ accurately reproduces the average [OH]_cal_ within 10 to 40% in the BB plumes with the required observations. The corresponding budget closure analysis further confirms that the model successfully reproduces observed P(HO*_x_*) and OHR_VOC_ within 20% for the five BB plumes up to a physical age of 5 hours. These results indicate that the MCM mechanism is sufficient for modeling OH reactions when initialized with HONO and ~90 VOC masses mostly measured by proton-transfer-reaction mass spectrometry (PTR-MS) in highly polluted plumes.

The MCM_GCVOC_ and GEOS-Chem, both using a reduced VOC representation, predict [OH]_model_ within 12 ± 5% against each other across the five BB plumes. However, their OH concentrations are 20 to 40% higher than those predicted by MCM_BBVOC_. Because both MCM_GCVOC_ and GEOS-Chem include and reproduce the major radical contributors except for diacetyl, we attribute their higher [OH]_model_ to missing OH sinks in their VOC initializations. Both MCM_GCVOC_ and GEOS-Chem underestimate the observed OHR_VOC_ by 46 ± 9% in the first 5 hours of aging, confirming recent findings (*9*, *17*). Key contributors to OHR_VOC_ are acrolein, 1,3-butadiene, and various furanoids, which together account for 75 ± 20% of the “missing” OHR_VOC_ in the fresh BB plumes and should be prioritized in future model development. A more detailed breakdown of BB OHR_VOC_ profile can be found in our previous work focused on western US BB plumes (*17*).

### Model performance for individual VOCs

We then assess how OH interacts with VOCs and evaluate how differing VOC assumptions in current models affect predictions. Subsequent sections expand this analysis to ozone, PAN, and other gas-phase organic nitrates (OrgN). As indicated by OHR_VOC_, we show large total VOC variability across BB plumes (Fig. 2C). Initial OHR_VOC_ varies by up to a factor of 3 across the BB plumes (table S3), driven by differences in fuel types, moisture content, combustion efficiency, and fire conditions (*11*, *12*, *52*). Total VOC decay rates also differ widely. For example, the Taylor Creek fire, with the highest elevated OH concentrations [>(10 ± 6) × 10^6^ molecules cm^–3^], shows rapid removal of total VOCs (e-folding lifetime of 3 hours). However, the Donnell and Bear Trap fires, with lower OH concentrations, exhibit slower decay (e-folding lifetimes: 12 hours).

We focus on individual VOCs whose dilution-corrected concentrations vary by more than 10% within the first 5 hours of physical aging (see section S2 for methodological details). This criterion identifies 37 VOC groups and/or masses of the ~90 individual VOCs, suggesting that their abundance changes are affected by OH-driven reactions rather than physical processes. Table S1 summarizes the model biases for these VOCs in different model runs. When evaluating model outputs against observations, MCM_BBVOC_ agrees with 7 of 12 primary VOC groups and/or masses within the combined measurements and sampling uncertainty (10 to 50%) on average. Specifically, MCM_BBVOC_ agrees with observed concentrations within 10% for xylenes; 10 to 30% for 2-butenal, cresol, phenol, and furanoids (except for 2,5-dimethylfuran and 3-methylfuran); and 40 to 50% for 1,3-butadiene and monoterpenes on average across the five selected BB plumes. The disagreements exceed 50% for other primary VOCs, including isoprene, 2,5-dimethylfuran, guaiacol, syringol, and sesquiterpenes. However, these discrepancies likely arise not only from errors in MCM_BBVOC_ but also from limitations in current PTR-MS measurements, including uncharacterized fragmentation and unavailable calibrations for oxygenated and terpenoid compounds (*52*). Overall, better speciation and quantification of these species in BB smoke are needed to further resolve model-observation biases of these reactive VOCs in future studies.

For the 15 secondary VOC groups and/or masses evaluated, MCM_BBVOC_ agrees with observations within 10% for seven species, including formaldehyde, acetaldehyde, methyl ethyl ketone (MEK), maleic anhydride, methacrolein + methyl vinyl ketone, methylglyoxal, and acrolein. The agreements between MCM_BBVOC_ and observations are within 10 to 30% for lumped C ≥ 3 aldehydes, glyoxal, hydroxyacetone, benzaldehyde, 3-methylfuran, and acetic acid + glycolaldehyde. The only exceptions are formic acid and diacetyl, which show systematically low biases (>50%) in all simulations due to missing precursors and PTR-MS uncertainties, respectively (see section S3 for more details).

MCM_GCVOC_ and GEOS-Chem show slightly lower and more degraded VOC abundances compared to MCM_BBVOC_. However, the three models agree with each other within ±15% for 23 of 28 comodeled VOC groups and/or masses within 5 hours of physical aging, with exceptions including monoterpenes, cresol, xylenes, MEK, and benzaldehyde (section S3). Notably, the modeled formaldehyde agrees with observations within 10% in three simulations (fig. S3). The agreement indicates a substantial improvement over previous evaluations using the GEOS-Chem CTM, which underestimated formaldehyde by a factor of 2 to 4 in fire-affected environments (*6*). The improvement could be attributed to the inclusion of ethene and acetylene chemistry (*53*), which, according to the MCM_BBVOC_, together account for ~20% of formaldehyde production in the first 5 hours of physical aging within the studied BB plumes. Overall, our results show that the current models reproduce most individual VOCs within instrument uncertainties, but important gaps remain for highly reactive or poorly constrained species (e.g., sesquiterpenes).

### Ozone abundance, formation, and sensitivity to precursors

**Fig. 3.**
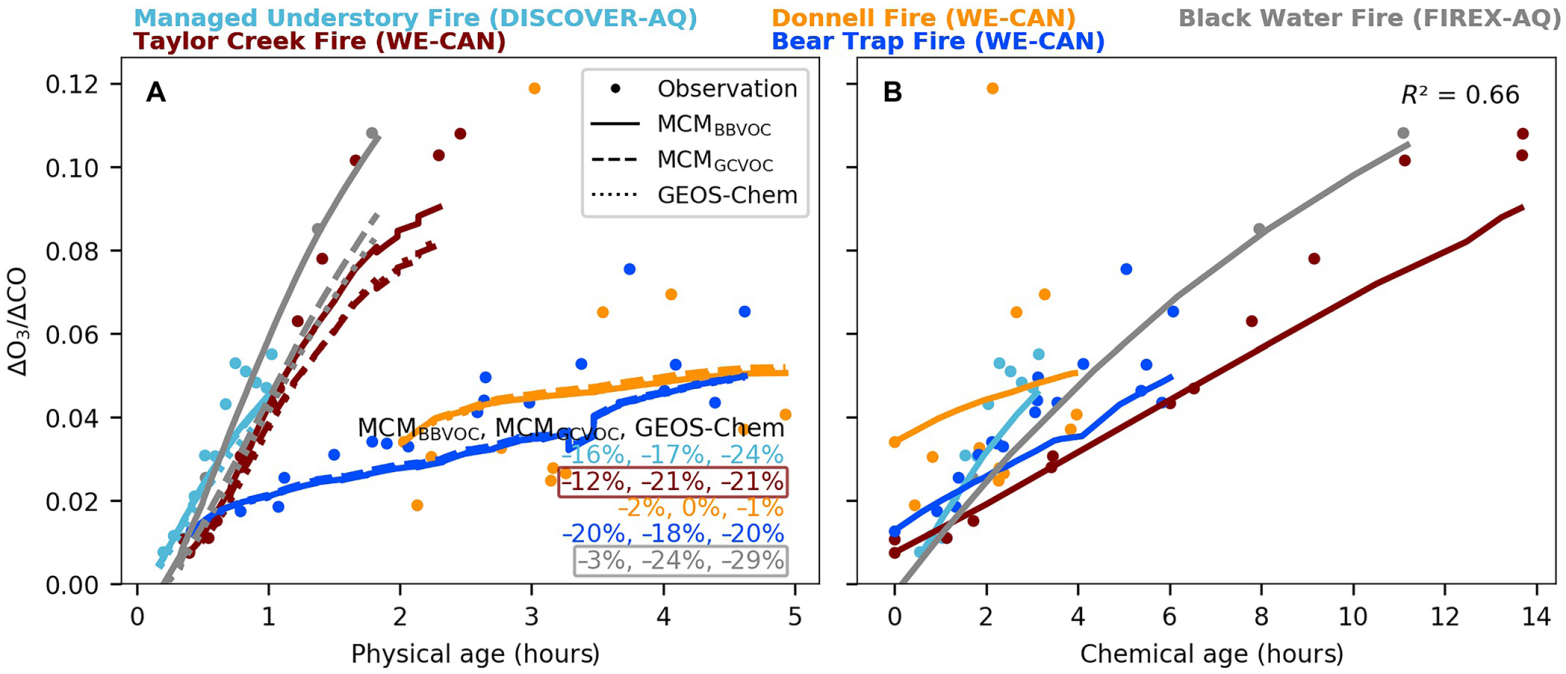
Evolution of ΔO_3_/ΔCO and its relationship with chemical age in BB plumes. (**A**) Evolution of ΔO_3_/ΔCO [parts per billion by volume (ppbv)/ppbv] within 5 hours of physical aging, colored by different BB plumes. Observations are shown in circles, and model results are shown by lines (MCM_BBVOC_ as solid lines; MCM_GCVOC_ as dashed lines; GEOS-Chem as dotted lines). The inset shows the corresponding NMB between models and observations. We note that the Taylor Creek and Black Water fires are distinguished by their higher OHR_NO*x*_:OHR_VOC_ values (>0.1, compared to 0.02 to 0.05 in the other three BB plumes; table S3). (**B**) Same ΔO_3_/ΔCO as (A) but plotted against chemical ages [defined by the ratio of OH exposure (Δ*t* × [OH]_model_) to the assumed ambient OH concentration]. Observations are shown as circles, and MCM_BBVOC_ results are shown as solid lines. The coefficient of determination (*R*^2^) between chemical age and observed ΔO_3_/ΔCO is 0.66.

Figure 3A shows rapid ozone formation in fresh smoke from five different BB plumes. Observations show that an average ozone normalized excess mixing ratio (ΔO_3_/ΔCO) of 4.4 ± 0.7% (range: 3.4 to 5.4%) within the first 5 hours of physical aging. The net ozone production rates [P(O_3_), as reflected by ΔO_3_/ΔCO per hour] vary by a factor of 4 across the five BB plumes (3.6 ± 1.8% hour^–1^; range: 1.4 to 5.8% hour^–1^). Across these BB plumes, those with higher initial OHR_NO*x*_:OHR_VOC_ ratios (an ozone sensitivity indicator) tend to have higher P(O_3_). P(O_3_) peaks within the first 1 to 2 hours, indicating that photochemistry is strongest during the period (fig. S4). Specifically, the first-hour P(O_3_) averages 4.5 ± 1.5% hour^–1^ (range: 1.9 to 7.1% hour^–1^) across the five BB plumes, with the P(O_3_) of other plume ages summarized in table S2.

Comparing our results with previous literature to elucidate ΔO_3_/ΔCO trends is complicated by several factors inherent in the earlier studies. First, many plumes in previous datasets were observed only once rather than at multiple stages of aging. Second, the fires often had poorly documented fuel types and weather conditions. Third, some past far-field measurements (≥2 days old) were made over 1000 km downwind of fire sources, where other sources could easily affect both the background and plume measurements (*54*). The same complications apply to ΔPAN/ΔCO as discussed later. Thus, direct comparisons with the literature are not provided.

The MCM, when initialized with HONO and ~90 VOCs (MCM_BBVOC_), predicts observed ΔO_3_/ΔCO with a negative bias of within 20% within 5 hours of physical aging, indicating no major gaps in current gas-phase ozone chemistry, confirming the findings in Xu *et al.* (*8*). Together, these results further infer that heterogeneous chemistry likely plays a minor role in ozone loss in fresh BB plumes (*8*, *47*). Initializing the model with fewer VOCs (MCM_GCVOC_ and GEOS-Chem) yields ΔO_3_/ΔCO predictions similar to MCM_BBVOC_ (within 10%) for most BB plumes. However, the consistency does not extend to the Taylor Creek and Black Water fires. These two BB plumes are distinguished by their higher OHR_NO*x*_:OHR_VOC_ values (>0.1, compared to 0.02 to 0.05 in the other three BB plumes; see table S3), indicating a greater limitation of ozone production by VOC availability. As a result, both MCM_GCVOC_ and GEOS-Chem predict ΔO_3_/ΔCO values that are 10 to 30% lower than observations and MCM_BBVOC_ in the two plumes. Further radical analysis of the two BB plumes reveals that the higher and more accurate ΔO_3_/ΔCO predictions by MCM_BBVOC_ are due to 10 to 25% higher XO_2_ (= HO_2_ + RO_2_) compared to MCM_GCVOC_ and GEOS-Chem, resulting in 10 to 60% higher P(O_3_) (fig. S5). Among the most important XO_2_ precursors in MCM_BBVOC_, half of the top 10 HO_2_ precursors and 4 of the top 10 RO_2_ contributors are absent from the GEOS-Chem mechanism used here, including furanoids, 1,3-butadiene, diacetyl, acrolein, and catechol.

Another key characteristic for understanding ozone production is its sensitivity to its precursors, NO*_x_* and VOCs. Xu *et al.* (*8*) found that plumes aged less than 12 hours generally fell into the NO*_x_*-limited regime in temperate forest plumes, based on the observation that the rate of self-radical removal exceeds the rate of radical removal by NO*_x_*. We revisit the sensitivity of ozone to VOCs and NO*_x_* using four additional photochemical indicators: (i) the ratio of formaldehyde to nitrogen dioxide (FNR), (ii) the relationship between FNR and NO, (iii) the ratio of OHR_NO*x*_ to OHR_VOC_ (nondimensional reactivity θ), and (iv) the ratio of self-radical removal to radical removal by NO*_x_* (*L*_RO*x*_:*L*_NO*x*_, where RO*_x_* = XO_2_ + OH). Among these indicators, all are available from our model simulations, but only the first three could be directly observed. Each indicator is chosen for either its scientific robustness or ease of accessibility, with details summarized in table S4.

**Fig. 4.**
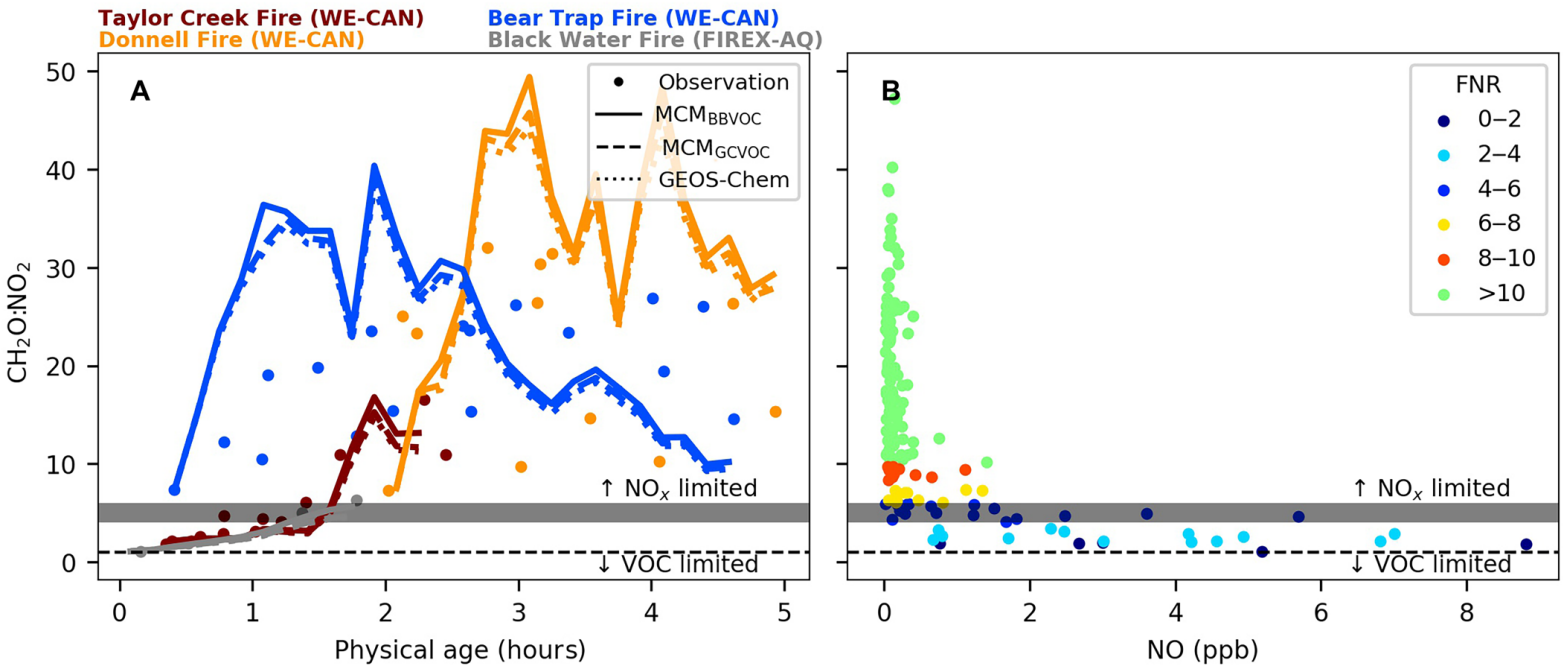
FNR evolution and its NO-dependent sensitivity in BB plumes. (**A**) Time series of the ratio of FNR (or CH_2_O:NO_2_). Observations are shown as circles, colored by different BB plumes. Also shown are model results from MCM_BBVOC_ (solid lines), MCM_GCVOC_ (dashed lines), and GEOS-Chem (dotted lines). (**B**) Sensitivity of FNR values to NO concentration. Observations are shown as circles, colored by different FNR ranges. Horizontal lines show the traditional (FNR = 1) and proposed thresholds (FNR = 4 to 6) for ozone sensitivity. ppb, parts per billion.

Figure 4 presents the time series of FNR and its response to NO concentrations in BB plumes within the first 5 hours of physical aging. Traditionally, plumes with FNR > 1 are considered NO*_x_*-limited regime, and those with FNR < 1 are VOC-limited (or transitional) (*55*). In the studied BB plumes, all FNR values exceed one, suggesting a NO*_x_*-limited regime. However, because the conventional FNR threshold was developed for urban environments, it may not accurately capture the chemical dynamics in more polluted or complex settings, including BB plumes (*55*, *56*). In such environments, a higher FNR threshold may be necessary due to the varying relationship between formaldehyde and its VOC precursors (*56*).

To establish a FNR threshold suitable for smoke-affected scenarios, we leverage the dynamic relationship between FNR and NO following Nussbaumer *et al.* (*57*), where sensitive changes in FNR with increasing NO indicate NO*_x_*-limited regimes and insensitive changes indicate VOC-limited regimes (with details provided in table S4). Figure 4B shows the relationship between FNR and NO concentration in BB smoke. To determine the FNR threshold, we use multiple statistical approaches, including *K*-means clustering, derivative-based slope thresholding (magnitude change ≥ 1), and piecewise linear regression. The statistical analyses identify an FNR threshold of 4 to 6, showing that FNR remains unresponsive to NO variations below this range in BB smoke. Using this threshold, we conclude that even the VOC-rich BB plumes could fall into a VOC-limited regime during the initial 2 hours of physical aging. In this VOC-limited regime, ozone production peaks because the near-field environment is chemically “intense” (Fig. 1), allowing net ozone formation before NO*_x_* depletion leads to a more NO*_x_*-limited regime.

**Fig. 5.**
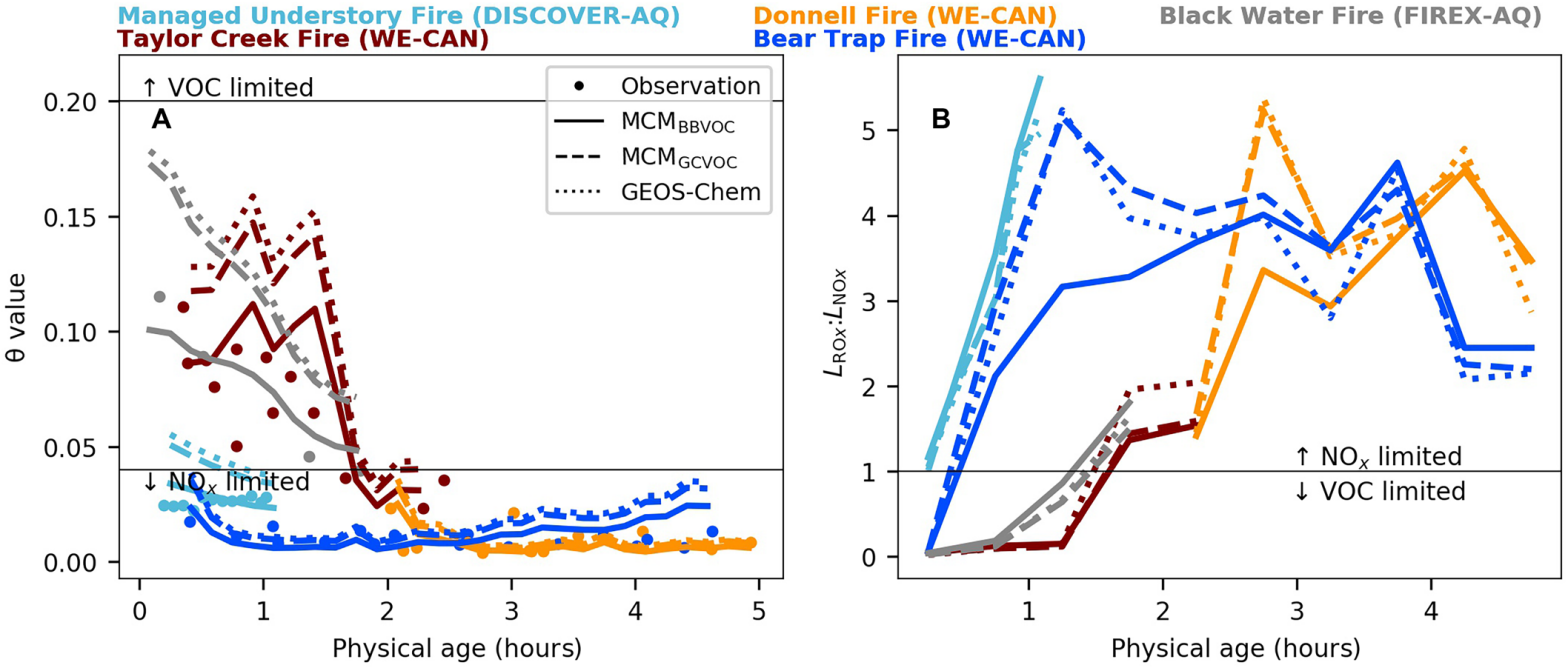
Time series of θ and *L*_RO*x*_:*L*_NO*x*_ in five BB plumes. (**A**) The ratio of OH reactivity from NO*_x_* to that from VOCs (θ). The horizontal black line represents the threshold where θ = 0.2 and θ = 0.04 (adjusted in this work). The observations are shown in circles, colored by different BB plumes. Also shown are model results from MCM_BBVOC_ (solid lines), MCM_GCVOC_ (dashed lines), and GEOS-Chem (dotted lines). (**B**) The ratio of radical self-reaction to reaction with NO*_x_* (*L*_RO*x*_:*L*_NO*x*_) in MCM_BBVOC_. The horizontal black line represents the threshold where *L*_RO*x*_:*L*_NO*x*_ = 1.

We further validate the above conclusions using two additional photochemical indicators: the nondimensional reactivity θ and the ratio of radical loss rates *L*_RO*x*_:*L*_NO*x*_ (Fig. 5). *L*_RO*x*_ represents the loss of RO*_x_* through self-reactions, while *L*_NO*x*_ is the loss of NO*_x_*. The θ indicator reflects whether NO*_x_* or VOCs dominate the OH loss pathway, while *L*_RO*x*_:*L*_NO*x*_ reflects the competition between peroxide formation (RO*_x_* self-reactions) and NO*_x_* as the primary RO*_x_* sink (*55*, *58*). Plumes are considered VOC-limited (or transitional) when the NO*x*-driven radical termination channel is significant, diagnosed by either θ > 0.04 (adjusted by the dynamic relationship between θ and NO) or *L*_RO*x*_:*L*_NO*x*_ < 1. Conversely, plumes are deemed NO*_x_*-limited when VOC dominates the OH loss pathway (θ < 0.04) or radical-radical reactions govern radical chain termination (*L*_RO*x*_:*L*_NO*x*_ > 1). A previous modeling study suggested that the *L*_RO*x*_:*L*_NO*x*_ should be adjusted to ~1.4 when more comprehensive NO*_x_* sinks were considered (*59*). However, changing the *L*_RO*x*_:*L*_NO*x*_ threshold does not change the conclusion we gained in this work.

Figure 5 shows that the observed θ could be >0.04 and the MCM_BBVOC_-calculated *L*_RO*x*_:*L*_NO*x*_ is <1 within the first 2 hours of physical aging, confirming that at least two of five BB plumes initially reside in a VOC-limited (or transitional) regime in the fresh smoke. As plumes age, a rapid transition occurs from a VOC-limited regime to a NO*_x_*-limited regime, likely driven by the rapid depletion of NO*_x_* and its short lifetime within BB plumes, facilitating the shift to a NO*_x_*-limited regime (*44*). The dynamic shift was also reported in other studies based on observation-constrained model simulations (*31*, *59*). The consistent results among these indicators and the previous modeling study support the robustness of our proposed FNR threshold of 4 to 6 in BB plumes. However, the proposed threshold should be viewed as an approximate indicator rather than an absolute boundary due to the widely varying characteristics of individual BB plumes.

The box models, using different mechanisms and VOC initializations, consistently align with observed FNR within 20% and modeled *L*_RO*x*_:*L*_NO*x*_ within 25% of each other. The consistency suggests that MCM and CTMs generally reproduce the ozone sensitivity observed in BB plumes. However, notable discrepancies occur in specific cases: the second hour of the Bear Trap Fire and the third hour of the Donnell Fire, where MCM_GCVOC_ and GEOS-Chem predict *L*_RO*x*_:*L*_NO*x*_ values 40% higher than those predicted by MCM_BBVOC_. The deviations suggest that current CTMs incorrectly predict a less VOC-limited regime in certain environments, further confirming the need for improved VOC representation in CTMs.

In addition, MCM_BBVOC_ agrees with observed θ within 10% across the five BB plumes. However, the CTM-based θ are biased high due to underestimated OHR_VOC_ (Fig. 2), which results in up to a 60% higher prediction of θ in both MCM_GCVOC_ and GEOS-Chem. Similarly, while *L*_RO*x*_:*L*_NO*x*_ can reveal ozone production regimes, it relies on specialized modeling analysis. By comparison, NO_2_, formaldehyde, and comprehensive speciated VOCs are relatively easy to measure with existing monitoring techniques, making FNR and θ useful field tools that use observations only for assessing ozone photochemistry in BB environments.

### Revisiting PAN and gas-phase OrgN

Oxidation products of BB VOCs rapidly react with NO*_x_* to form other reactive oxidized nitrogen species (NO*_y_*), including PAN, pNO_3_, and gas-phase OrgN (excluding PAN and peroxy propionyl nitrate (PPN). Juncosa Calahorrano *et al.* (*60*) detailed observed NO*_y_* partitioning during the first 4 hours of daytime smoke evolution using comprehensive measurements from WE-CAN. Building on the same observations, Peng *et al.* (*29*) evaluated modeled NO*_x_* fate using MCM, with a fewer number of VOCs compared to MCM_BBVOC_ (~40 versus 90 VOCs). Here, we revisit and extend the previous work using a broader range of BB VOCs to explore potential weaknesses in NO*_y_* partitioning within both MCM and GEOS-Chem mechanisms. Notably, this is the first time the GEOS-Chem mechanism has been assessed for plume-level BB chemistry.

**Fig. 6.**
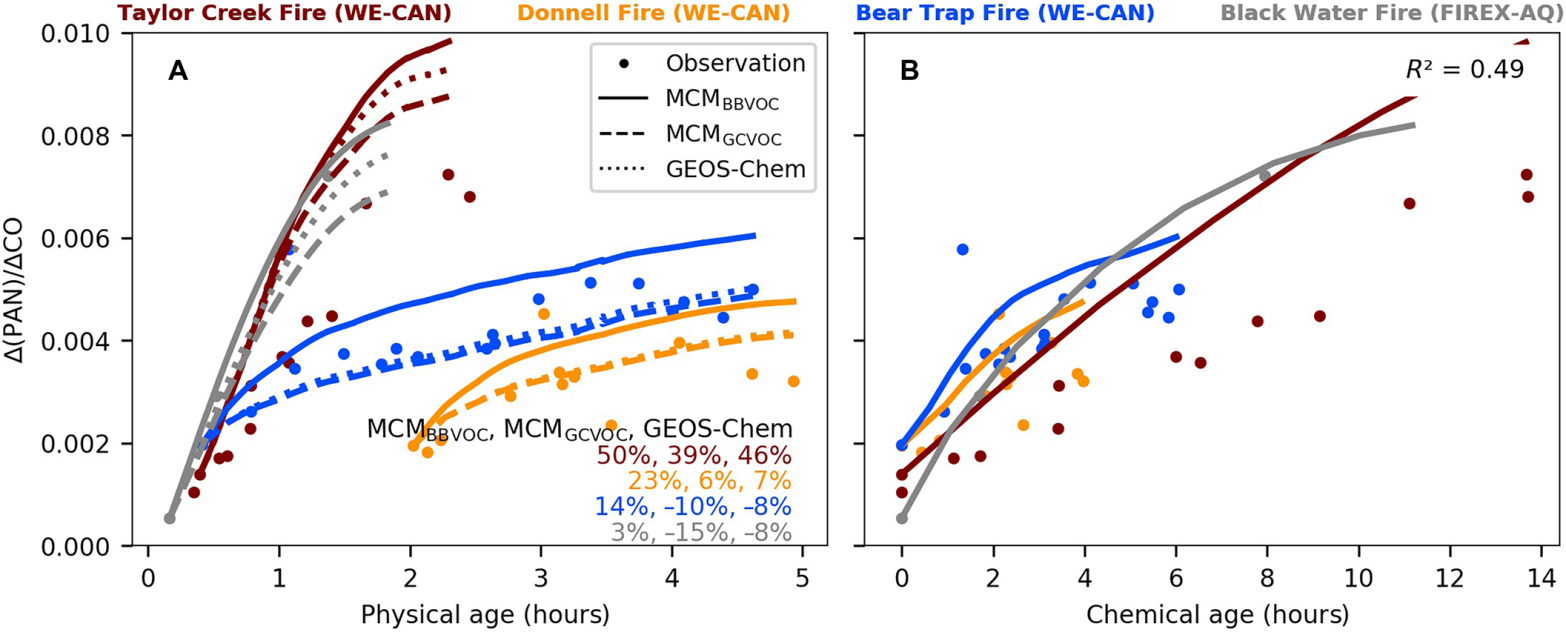
Evolution of ΔPAN/ΔCO and its relationship with chemical age in five BB plumes. (**A**) Evolution of ΔPAN/ΔCO (ppbv/ppbv) within 5 hours of physical aging, colored by different BB plumes. Observations are shown in circles, and model results are shown by lines (MCM_BBVOC_ as solid lines; MCM_GCVOC_ as dashed lines; GEOS-Chem as dotted lines). The inset shows the corresponding NMB between models and observations. (**B**) Same PAN/ΔCO as (A) but plotted against chemical ages [defined by the ratio of OH exposure (Δ*t* × [OH]_model_) to the assumed ambient OH concentration]. Observations are shown as circles, and MCM_BBVOC_ results are shown as solid lines. The *R*^2^ between chemical age and observed ΔPAN/ΔCO is 0.49.

Figure 6 shows the evolution of ΔPAN/ΔCO as a function of both physical and chemical ages in four BB plumes where measurements were made. According to model diagnostics, PAN loss via chemistry and thermal decomposition is minimal in studied fresh BB plumes with plume temperature ranging 0° to 20°C. Thus, we conclude that the observed ΔPAN/ΔCO is mostly driven by the production. Similar to ozone, we show PAN production peaks within the first hour and then stabilizes as plumes age, with ΔPAN/ΔCO showing an average of 0.35 ± 0.03% (range: 0.30 to 0.39%) across four studied plumes within the first 5 hours of aging. However, the magnitude of PAN production is an order of magnitude lower than that of ozone production. The net production rates of PAN [P(PAN); reflected by ΔPAN/ΔCO per hour] are estimated at 0.27 ± 0.14% hour^–1^ (range: 0.09 to 0.47% hour^–1^), and P(O_3_) of the same BB plumes is 3.6 ± 1.8% hour^–1^.

MCM_BBVOC_ overestimates observed ΔPAN/ΔCO by within 20% in three of four BB plumes and by 50% in the Taylor Creek Fire. Notably, MCM_GCVOC_ and GEOS-Chem show similar positive biases in the Taylor Creek Fire, indicating that the issue is not specific to mechanism configuration. Including certain key PAN precursors (e.g., diacetyl) in GEOS-Chem would increase predicted PAN by 10 to 30%, worsening the model performance. Thus, further investigation into the overestimated PAN is needed, as similar positive biases are found in all three models in the Taylor Creek Fire.

Given that PAN loss is minimal in studied BB plumes, the positive model bias is likely due to overestimated PAN formation. Although MCM_BBVOC_ accurately reproduces key VOC precursors within 20% (table S1)—including acetaldehyde (52 ± 3% of total PAN production across five BB plumes in MCM_BBVOC_), diacetyl (24 ± 4%), and methylglyoxal (15 ± 6%)—the model could underestimate NO*_x_* sinks, particularly for the Taylor Creek Fire. One such sink is the pathway leading to nitrate particle formation, which is discussed in the following texts. By underestimating these sinks, more NO*_x_* remains available to react with peroxyacetyl radicals, leading to enhanced PAN production in the model. Similar conclusions were drawn by Peng *et al.* (*29*), indicating the need to refine NO*_x_* sink representations to improve PAN prediction accuracy in fresh BB smoke.

Peng *et al.* (*29*) attributed the underestimated NO*_x_* sinks to a constant missing source of 70 parts per thousand (ppt) of XO_2_, which would artificially convert NO*_x_* into OrgN. However, this assumption might be flawed, as it doubled the modeled XO_2_ levels in BB plumes, contradicting more recent findings (*17*, *28*). For example, Permar *et al.* (*17*) and this work (Fig. 2C) show that MCM could account for more than 90% of the initial OHR_VOC_ in fire emissions, reducing the likelihood of notable unidentified VOCs producing excess XO_2_. Furthermore, Lindsay *et al.* (*28*) indicated that MCM could even overestimate observed XO_2_ by 30% in BB plumes aged from hours to days, further challenging the need to introduce additional XO_2_ sources as proposed by Peng *et al.* (*29*).

The underestimated NO*_x_* loss in the plume is not unexpected. WE-CAN observations suggested that three NO*_x_* sinks play an important role: PAN (37%), pNO_3_ (27%), and OrgN (22%), collectively accounting for ~86% of NO*_y_* in the fresh BB smoke (*60*). However, pNO_3_ formation is not included in the gas-phase simulations of this work. If incorporated correctly, it should help resolve the model NO*_x_* sink issue (*61*). Sensitivity tests suggest that we would need to assume the pNO_3_ formation to be four times that of HNO_3_ in MCM to correct the positive model bias in PAN in Taylor Creek Fire. Further investigation into pNO_3_ sinks, such as photolysis (*49*), remains necessary, as these processes are uncertain. Below, we focus our analysis on the gas-phase OrgN.

We find that MCM_BBVOC_ agrees well with observed ΔOrgN/ΔCO within 20% (fig. S6), which suggests that OrgN abundance alone could not explain the positive model bias in PAN in Taylor Creek Fire. However, the factional contribution of OrgN species is different between the model and observation (fig. S7). WE-CAN observations reveal that, on average, most OrgN in fresh BB plumes consists of lower molecular weight nitrates (fig. S7): About 45% are C_2_ to C_4_, and 40% are C_5_ to C_7_ nitrates. The remaining 15% includes larger nitrates (C_8_ to C_17_). This distribution of OrgN composition is consistent across individual WE-CAN BB plumes, although we note that the observed OrgN is highly uncertain due to the lack of calibration (*60*, *62*). Nevertheless, MCM_BBVOC_ shows a much larger fraction of C_2_ to C_4_ nitrates (>80% versus 45%) and a smaller fraction of C_5_ to C_7_ nitrates (15% versus 45%) than in observations. MCM_GCVOC_ performs slightly worse, underestimating ΔOrgN/ΔCO by 30% due to incomplete VOC representation. GEOS-Chem performs the worst, failing to capture both the observed abundance and variability (fig. S6), reflecting both the limited number of OrgN species (<10 versus >200 in MCM) and its simplified chemical treatment; for example, using the same RO_2_ + NO branching yields shared across lumped RO_2_ classes, using assumed first-order loss rates, and largely omitting secondary and/or multigeneration nitrate formation pathways. The models’ inability to accurately replicate the observed OrgN composition indicates a knowledge gap in the representation of OrgN yields and formation pathways in existing chemical mechanisms.

## DISCUSSION

### Summary

We assess current understanding of photochemistry in BB plumes with a physical age less than 5 hours and identify key underrepresented processes that limit accurate modeling of photochemical evolution. We calculate plume-center OH radical concentrations ([OH]_cal_) using changing ratios of three selected VOCs and CO. The three VOCs include furan, furfural, and furanone, which are chosen because they are primarily consumed by OH oxidation, have minimal secondary production, and have lifetimes suitable for our study. We carefully validate the [OH]_cal_ method by comparing different VOC:CO pairs and using modeled concentrations as pseudo-observations under idealized conditions. We then compare average [OH]_cal_ to modeled OH concentrations ([OH]_model_) from the best-guess simulation, which uses the MCM initialized with measured HONO and ~90 VOCs. We find that the [OH]_model_ agrees with [OH]_cal_ within 10 to 40% for plumes with the required observations. The model bias increases for older plumes, but it is likely due to higher measurement requirements for detecting VOC decay, nonideal sampling conditions, and increased plume dilution. Combined with an OH budget closure analysis, which shows that the model accurately reproduced both the sources and sinks of OH within 20%, we conclude that there are no major knowledge gaps in the established gas-phase chemistry regarding OH prediction in BB plumes.

We track the temporal evolution of OH with reasonable precision across the first 5 hours of aging at BB plume centers. We find notable variability in OH concentrations across the studied plumes; however, concentrations are consistently elevated in fresh BB plumes compared to typical ambient OH concentrations (~1.5 × 10^6^ molecules cm^−3^). We show OH concentrations peak at 5 to 20 × 10^6^ molecules cm^−3^ in the first 1 to 2 hours (averaged using [OH]_model_) and return to ambient OH concentrations within the next 3 to 5 hours. High HONO levels drive the OH enhancements during the first hour, while OVOC photolysis and ozone become more important OH sources, as physical age increases. We use chemical age as a metric to quantify the photochemical oxidation, which is the equivalent time required at ambient OH concentrations to achieve the same integrated OH exposure. We show that chemical age is three to four times physical age within 2 hours of physical aging; thus, 1 hour of physical aging in these fresh plumes is equivalent to 3 to 4 hours of photochemical reactions under typical ambient environments. As BB plumes age, the chemical-to-physical age ratio decreases, transitioning from 4 to 1 between the second hour of physical aging and the more aged smoke, a shift driven by HONO depletion and atmospheric dilution.

We estimate production rates of O_3_ and PAN and their relationship with chemical age across five studied BB plumes. We find that OH evolution drives the formation of secondary pollutants. Specifically, we show rapid production of ozone and PAN during the first hour of plume aging [P(O_3_): 4.2 ± 1.5% hour^–1^ and P(PAN): 0.4 ± 0.1% hour^–1^]. As the plumes age, both observed P(O_3_) and P(PAN) slow down. Notable variability in PAN and ozone formation is found across selected BB plumes, with mean production rates varying up to fivefold [P(O_3_): 1.4 to 5.8% hour^–1^ and P(PAN): 0.1 to 0.5% hour^–1^] within 5 hours of physical aging. About 40 to 60% of the variability in the decay of total VOC and the production of PAN and ozone, across plumes and fuel types, could be explained by the chemical age of the plumes, suggesting that chemical age provides a unifying diagnostic for near-field plume chemistry across fires.

We cross-validate photochemical indicators and assess ozone dependence on NO*_x_* and VOCs in fresh BB plumes. We show that traditional thresholds of photochemical indicators, developed for urban environments, differ from those applicable in smoke-affected scenarios, including the ratio of FNR. By examining their dependence on NO*_x_* in five fresh BB plumes, we infer an approximate FNR range (~4 to 6) separating transient VOC-limited (or transitional) and NO*x*-limited behaviors in these fresh plumes. Using multiple photochemical indicators, we find that BB plumes could remain in a VOC-limited regime for the first 2 hours of physical aging but shift to NO*_x_*-limited further downwind.

We conduct and compare three box model simulations to assess the impact of varying chemical mechanism complexities and VOC representations. When initialized with the same reduced VOC set (<40 species), models produced OH, VOC, O_3_, and PAN that agreed within ~15% over the first 5 hours, regardless of whether we use the near-explicit MCM or the simplified CTM mechanism. However, the model initialized with explicit BB VOCs (represented by ~90 species) shows more differences (>20%) compared to CTM-VOC–initialized models, suggesting that VOC representation is the major driver of model differences under the tested BB plumes.

We further compare model simulations with observations to evaluate model performances. Three model simulations predict the concentrations of most VOCs, including formaldehyde, within the instrument uncertainties of 15 to 50%. For other key atmospheric compounds including OH, ozone, and OrgN (excluding PAN and PPN), the model using explicit BB VOCs generally outperforms the other two, agreeing with observations within 20%. As the MCM model, based solely on gas-phase chemistry, sufficiently reproduces the observed OH and ozone production, we infer that heterogeneous losses of OH and ozone are likely minor in wildfire plumes when photolysis rates are constrained by observations. We find in one of four BB plumes examined here that even the best model could overestimate PAN by 60%, likely due to underestimated NO*_x_* loss pathways. The other two models, which are initialized with CTM VOCs, show notable model biases. They overpredict OH concentrations by up to ~50% across the three tested BB plumes, underpredict ozone by 20 to 30% in two VOC-limited BB plumes, and underpredict OrgN by ~50% in three WE-CAN plumes with available measurements. The model biases stem from the absence of key VOCs in the CTM that are crucial for consuming OH and acting as precursors for ozone and OrgN. Important missing compounds include acrolein and various furanoids, which consistently influence OH, ozone, and OrgN. In addition, 1,3-butadiene, diacetyl, and catechol are also identified as important contributors to the dynamics of either OH, ozone, or OrgN. Their incomplete representation remains a key limitation in the current mechanisms for smoke-affected environments.

### Limitations

We acknowledge several caveats to our findings. First, uncertainties of uncalibrated VOCs measured by PTR-MS can be 50%, while uncertainties in chemical ionization mass spectrometer (CIMS) measurements of OrgN can be even 100%. These discrepancies are largely due to unknown isomers and/or fragments and unavailable calibrations. Second, plume dilution rates can vary over time and across the plume, due to varying turbulence and out-of-plume concentrations. We use a single coefficient for dilution in models, which may not effectively capture these variations in aged smoke. Third, we focus on chemistry at the plume center, but the plume chemistry may change in the peripheral regions; future investigations should explore edge chemistry for completeness. Fourth, while we find that gas-phase chemistry alone can explain most of the observed OH and ozone when photolysis rates are constrained by observations, we acknowledge that more explicit treatment of heterogeneous chemistry may be warranted when future laboratory or field experiments can better refine key uptake or reaction rates in BB related environments. Last, we note that while newer GEOS-Chem versions have added species such as furanoids, 1,3-butadiene, acrolein, and catechol (lumped), their oxidation pathways remain simplified, which suggests the need for continued mechanism development for BB plume chemistry in CTMs.

### Implication

Millions of people across the western US are affected by hazardous air quality conditions caused by wildfire smoke in the summer. However, predicting chemical evolution in BB-influenced environments is challenging. We identify current limitations in commonly used explicit and simplified chemical mechanisms. We find that the inclusion of specific VOCs in CTMs can improve model predictions in OH, ozone, and reactive nitrates. The results can help prioritize future developments of CTMs in predicting air pollution and its health impacts in BB-affected environments. We find that chemical age reduces the variability in the oxidation of total VOCs, ozone, and PAN across different BB plumes, indicating that differences in photochemical activity are key to understanding chemical evolution.

## MATERIALS AND METHODS

### Observational data

We chose five BB plumes sampled in three aircraft campaigns, focusing on the first 5-hour near-field chemical evolution (table S3). The physical age of plumes was estimated using transect-averaged wind speeds measured by the aircraft and the distance to the identified fire sources (*63*). The selected BB plumes include three research flights from the 2018 WE-CAN, one from the 2019 FIREX-AQ (Fire Influence on Regional to Global Environments and Air Quality), and one from the 2013 DISCOVER-AQ. All measurements were conducted during daylight hours (local time: 12:00 to 18:00).

These BB plumes were selected for their pseudo-Lagrangian sampling strategy, where the aircraft intercepted the same plume air mass from a known source multiple times through consecutive downwind transects. The setup allowed us to continuously track the evolution of the same air mass, making these data well suited for box modeling of chemical evolution (*64*). The same datasets have also been widely used in previous independent work (*14*, *17*, *18*, *29*, *63*). To further evaluate this approach, we calculated Δbenzene/ΔCO and showed that this ratio remained stable, varying by less than 10% on average over 5 hours, suggesting that the downwind samples had evolved from similar initial emissions (fig. S8). In addition, we used acetonitrile and CO_2_ to verify that the sampled points reflected the same combustion phase (i.e., flaming or smoldering). Nearly all data remained stable, with the exception of four outlier data points in the Bear Trap Fire, where the acetonitrile/ΔCO ratio deviated notably. Thus, we excluded these four points to ensure the robustness of our pseudo-Lagrangian dataset.

Our compilation of datasets offers a diverse range of fire conditions, spanning from the western to the southeastern United States, covering five states with various fuel types and plume sizes, from small fires burning across several acres to large fires exceeding 20,000 hectares. This diversity is also reflected by the initial chemical conditions measured by the aircraft. Specifically, initial CO mixing ratios range from 1 to 5 parts per million (ppm), partially reflecting varying levels of dilution before sampling by aircraft. The resulting initial OHR_VOC_ ranges from 54 to 142 s^–1^, and the initial OH reactivity from NO*_x_* (OHR_NO*x*_) from 1 to 16 s^–1^ (table S3). These large variabilities allow us to examine various real-world photochemical regimes and test the robustness of model chemistry. Additional plume data from other WE-CAN BB plumes with physical ages up to 18 hours were also used in the analysis, although these more aged data have higher uncertainty due to plume dilution and the difficulty in identifying their sources.

Table S6 summarizes the trace gas measurements and radiation measurements used in this work, including NO, NO_2_, HONO, PAN, ozone, CO, VOCs, and photolysis frequencies. All three aircraft campaigns deployed one or more instruments to measure VOCs. Here, we used the VOC observations mostly made by PTR-MS for its high temporal resolution (<1 s), ideal for measuring fresh plume transects that typically had strong spatial gradients even in narrow plumes (~1 to 10 km across; <2 min airborne measurements). Whenever applicable, isomeric fraction information derived from laboratory burning experiments was assigned to PTR-MS ion masses for speciation VOCs, which were used to compare to model outputs (*12*). For this work, we focused on the chemical evolution of the BB plume center, defined as the top 5% of CO observations of each transect.

### F0AM box model setup

To test our knowledge of photochemistry during the first few hours of plume aging, we used a simple box model framework rather than a full three-dimensional CTM, which inherently couples chemistry with uncertainties in emissions, plume rise, transport, and spatial resolution. We simulated the above five BB plumes in their first 5 hours of near-field chemical evolution using two atmospheric chemical mechanisms within the freely available F0AM box model (*64*): the updated MCM (version 3.3.1) and GEOS-Chem chemistry (“Fullchem” mechanism, version 13.3.0). The updated MCM, referred to simply as MCM for brevity, includes reactions for furans and key phenolic species from recent developments (*14*, *65*).

We conducted three box model simulations with different chemical mechanisms and VOC initialization profiles, reflecting various levels of chemical complexity (Table 1). Specifically, MCM_BBVOC_ initializes the MCM with ~90 VOC masses directly constrained by observations, offering a near-explicit chemical representation. GEOS-Chem uses its standard chemistry with 30 VOCs, commonly used in CTMs. We also performed an MCM_GCVOC_ run, which uses MCM but is initialized with standard GEOS-Chem species, resulting in 55 VOCs. The difference in VOC numbers between MCM_GCVOC_ and GEOS-Chem results from MCM’s explicit representation of certain lumped species. For example, while GEOS-Chem lumps all alkanes with four or more carbon atoms into a single category (lumped C ≥ 4 alkanes), MCM distinguishes these as 12 separate alkane species in this study.

**Table 1.** Summary of box model simulations.

Simulation	Description	# of initialized VOCs
MCM_BBVOC_	MCM chemical mechanism, with comprehensive BB VOCs	87
MCM_GCVOC_	MCM chemical mechanism, with CTM VOCs	55^*^
GEOS-Chem	GEOS-Chem chemical mechanism, with CTM VOCs	30

* Lumped VOCs in the GEOS-Chem such as lumped C ≥ 3 alkenes (PRPE), lumped C ≥ 4 alkanes (ALK4), lumped C ≥ 3 aldehydes (RCHO), xylenes (XYLE), and monoterpenes (MTPA) are explicitly represented in the MCM_GCVOC_ and MCM_BBVOC_. Details of the speciation of lumped VOCs are provided in table S7.

Initial conditions for simulations were established on the basis of observations closest to the fire for key compounds such as NO, NO_2_, HONO, ozone, CO, and CH_4_. The VOC initial concentrations in the model were either directly from aircraft measurements or were estimated using previously reported emission ratios scaled to the observed CO abundance when measurements were not available (*11*, *38*). Table S7 shows details of VOCs used to initialize the model experiments in this work.

Photolysis frequencies for model simulations were constrained through direct measurements whenever possible (table S8). For frequencies where direct measurements were unavailable, we calculated *J* values based on solar zenith angle, altitude, overhead ozone column, and albedo. The calculations used F0AM’s “hybrid” function (*64*), with references to lookup tables from the NCAR TUV v5.2 radiation model. In addition, other key model parameters such as pressure, temperature, and relative humidity were constrained by observations to ensure model fidelity.

Modeled NO and NO_2_ were determined using the F0AM’s FixNO*_x_* function, which maintains the model-calculated NO:NO_2_ ratio but constrains the total NO*_x_* (NO + NO_2_) level according to observations. The method is suitable considering that our observational step interval (4 to 10 min) is much shorter than the typical mid-day NO*_x_* lifetime (~hours) (*66*, *67*). The application of the FixNO*_x_* function mitigates the underestimation of NO*_x_* decay in the model, thereby improving the accuracy of ozone simulation (*29*, *68*).

We calculated dilution rates (*k*_dil_) using observed CO to account for background mixing as recommended by previous studies (*14*, *18*, *45*). The *k*_dil_ values were determined to achieve the best fit between modeled and observed CO. In our case studies—Taylor Creek, Bear Trap, Donnell, Blackwater, and Managed Understory fires—the *k*_dil_ values are 9 × 10^−4^, 5 × 10^−4^, 5 × 10^−4^, 1 × 10^−4^, and 2 × 10^−4^ s^−1^, respectively. We also conducted sensitivity tests for *k*_dil_ using long-lived primary VOCs such as benzene instead of CO, which confirmed the robustness of these *k*_dil_ values.

We then evaluated these three simulations with observed OH, VOCs, ozone, PAN, and other OrgN to assess how well each simulation represented photochemistry in heavily polluted environments. We also assessed the differences among these simulations and interpreted the results in terms of VOC representation (MCM_BBVOC_ versus MCM_GCVOC_) or chemical mechanisms (MCM_GCVOC_ and GC) and their implications for radical cycles and formation of OrgN and ozone. MCM_BBVOC_ was considered our best estimate and served as a reference for the other two simulations as previous mechanism comparison work (*69*, *70*).

## Supplementary Materials

### This PDF file includes:

Supplementary Text Figs. S1 to S8 Tables S1 to S8 References
