# Supplementary material for: Ozone photochemistry in fresh biomass burning smoke over the United States
Source: Sci Adv. 2026 Feb 6;12(6):eads2157. doi: 10.1126/sciadv.ads2157 (PMC12880535; doi:10.1126/sciadv.ads2157)
Supplement: Supplementary file 1 — Supplementary Text Figs. S1 to S8 Tables S1 to S8 References [file sciadv.ads2157_sm.pdf]

Science Advances
AAAS

Supplementary Materials for

Ozone photochemistry in fresh biomass burning smoke over the United States

Lixu Jin *et al.*

Corresponding author: Lixu Jin, lixu.jin@umontana.edu

Sci. Adv. **12**, eads2157 (2026)
DOI: 10.1126/sciadv.ads2157

This PDF file includes:

Supplementary Text
Figs. S1 to S8
Tables S1 to S8
References

S1 Calculation of radical production

We examined the budget of radical concentrations in fire plumes, focusing on their production rates ($P(HO_x)$; $HO_x = OH + HO_2$) and the VOC OH reactivity (reflecting the OH sink). Following the methodology outlined in Peng et al. (*45*), we calculated the $P(HO_x)$ by multiplying the precursor with its photolysis rate coefficient and summing these products. The radical precursors included HONO, ozone, and six OVOCs. Specifically, we corrected formaldehyde measurements in Peng et al. (*45*) which doubled the abundance in the previous analysis. We included four OVOCs not considered previously: glyoxal, methylglyoxal, glycolaldehyde, and 2,3-butanedione (diacetyl). We excluded ozonolysis due to its negligible impact compared to the considered photolytic sources. The photolysis of propanal, butanal, acetone, MEK, acrolein, methacrolein (MACR), MVK, hydroxyacetone, and furfural were excluded for the same reason. The impact of these exclusions on the radical production rate was less than 5% within 18 hours of aging.

S2 Dilution corrected mixing ratio

We calculated the normalized excess mixing ratio (NEMR) and dilution corrected mixing ratio using sampled plume-center concentration and corresponding background concentrations. The plume-center and background concentrations were estimated by the top and bottom 5 percentile of the sampled concentration across plumes, respectively. We used CO as a dilution tracer for compound X due to its dominance in primary fire emissions and its slow reaction rate with OH, which results in a lifetime of approximately one month at ambient OH concentrations. The details were shown in Equations 1 and 2.

$$NEMR = \frac{\Delta X}{\Delta CO} = \frac{X_{plume} - X_{background}}{CO_{plume} - CO_{background}} \quad (1)$$

$$Dilution\ corrected\ mixing\ ratio = \frac{\Delta X}{\Delta CO} \times \left(CO_{intial} - CO_{background}\right) \quad (2)$$

S3 Model comparison in specific VOCs

Model evaluation in MCM_{BBVOC}

MCM_{BBVOC} significantly underpredicts formic acid and diacetyl, with model biases exceeding 50% across all simulations. For formic acid, our recent study found many precursors could potentially contribute to the rapid formic acid formation in fresh smoke, including ketene–enol and dicarbonyl oxidation from aromatic and furanoid compounds and possible heterogeneous reactions (*71*). However, the exact chemistry of secondary productions is not well known and is still not fully represented in either MCM or GEOS-Chem chemical mechanisms.

For diacetyl, an important PAN precursor, MCM_{BBVOC} agrees with observations within 10% for the Taylor Creek fire but consistently underestimates its abundance by 80% in the other two fires where the measurements were reported (Donnell and Bear Trap fires). In the Donnell and Bear Trap fires, the modeled decay rates of diacetyl are considerably faster than observed, with e-folding lifetimes of 0.7 hours in MCM_{BBVOC} versus 2.3–2.6 hours in observations. We tentatively attribute the negative model bias to potential measurement uncertainties in PTR-MS. The fast modeled diacetyl decay rates suggest that the inaccuracies in observation-constrained photolysis rates might also contribute to the bias, since photolysis is the dominant loss process per MCM, accounting for >95% of its total loss rates. In either case, this highlights the need for better quantification of diacetyl and its photolysis, given its important role in $P(HO_x)$ and PAN production.

Model evaluation in MCM_{GCVOC} and GEOS-Chem

We provided a detailed model comparison for VOCs that agree with each other beyond 15%, including monoterpenes, cresol, xylenes, MEK, and benzaldehyde. Monoterpenes and cresol were predicted to be 15–30% lower in MCM_{GCVOC} and GEOS-Chem compared to MCM_{BBVOC} due to their higher OH concentration resulting from reduced VOC initializations. Xylenes were predicted to be 20% higher in GEOS-Chem compared to both MCM runs, attributed to higher $k_{OH+Xylenes}$ values used in GEOS-Chem compared to MCM and other recommended values (1.7 vs 1.1–1.3, in units of 10^{-11} cm^3 $molecules^{-1}$ s^{-1} when T=298K and 1 atm). MEK was predicted to be 20% higher in GEOS-Chem compared to MCM_{BBVOC} and MCM_{GCVOC} due to monoterpene oxidation pathways in GEOS-Chem, which accounted for 85–95% of MEK production. Removing this production pathway in GEOS-Chem reduced the model bias to within 10%. The reaction originally from RACM2 might result in MEK production being too high (*72–76*), potentially explaining MEK overestimation in low-to-no smoke conditions (*6*). Benzaldehyde was predicted to be ~30% lower in MCM_{GCVOC} and GEOS-Chem compared to MCM_{BBVOC} due to missing precursors in the CTM VOC representations, notably styrene, accounting for 90–95% of benzaldehyde's secondary source.

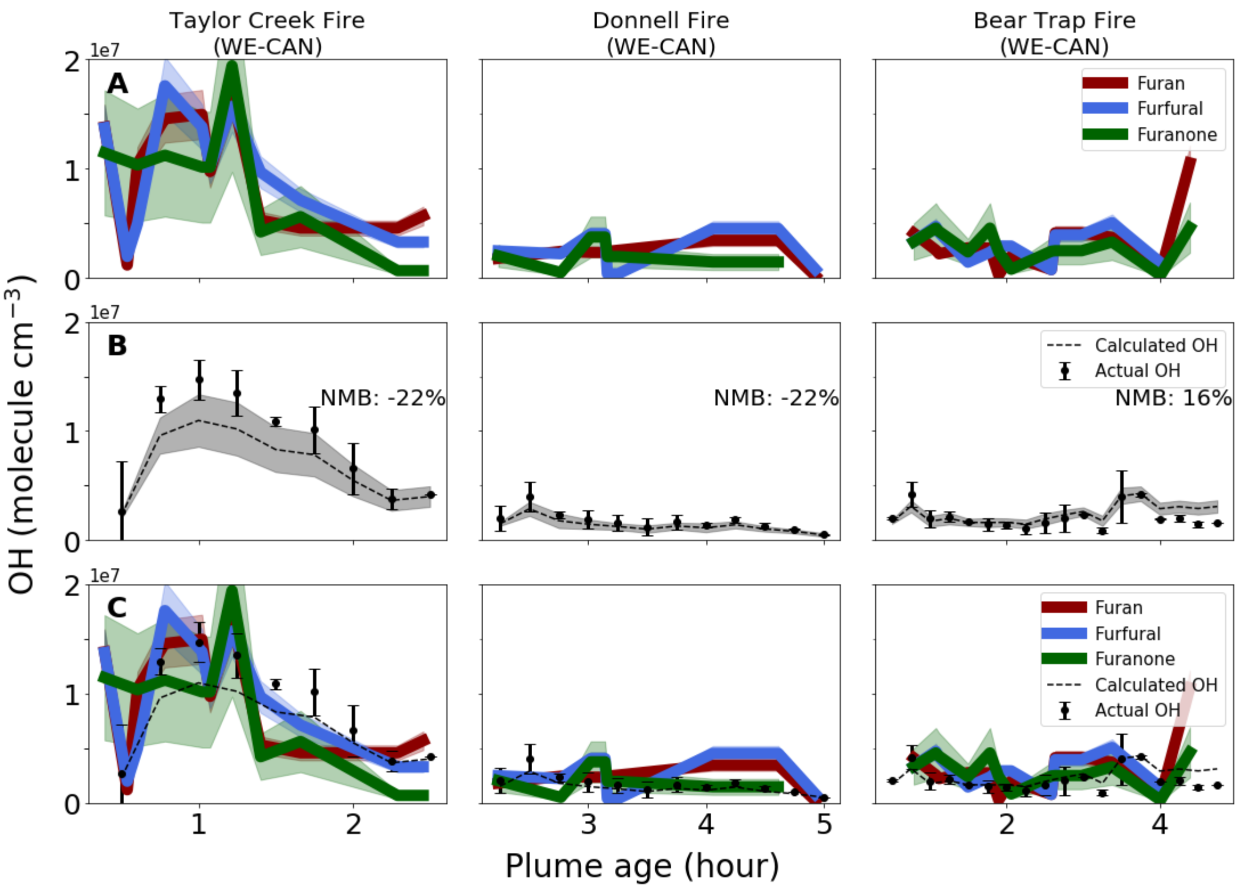

Fig. S1. Time series of median OH concentration. (a) Observed calculated OH estimations using selected VOCs, colored by different VOC-CO pairs and shaded by measurement uncertainty. Selected VOCs include furan (red), furfural (green), furanone (blue). (b) Averaged calculated OH concentrations and outputted OH concentrations from the MCM_{BBVOC}. Model results are binned to the nearest 15 min for better visualization. The dashed line shows the averaged calculated OH from the three selected VOCs, with shading showing the interquartile range of results in each bin. The error bar represents the actual OH outputted from the MCM_{BBVOC}, showing the interquartile range of results in each bin. (c) The combination of (a) and (b).

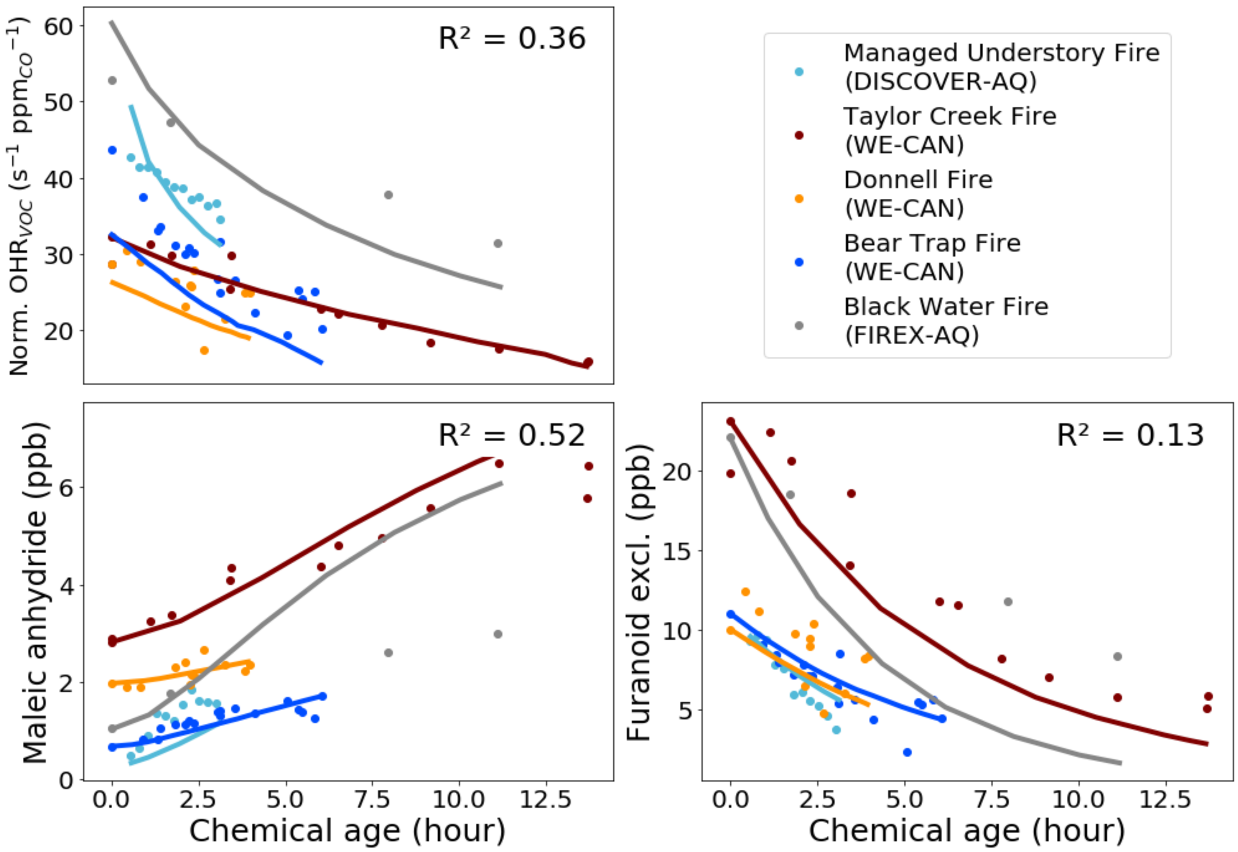

Fig. S2. Evolution of normalized VOC OH reactivity (OHR_{VOC}) (s^{-1} ppm_{CO}^{-1}) and dilution-corrected concentration of two VOC groups (ppb) against chemical age (defined by the ratio of OH exposure to the assumed ambient OH concentration). The two individual VOC groups include maleic anhydride and furanoids. Furanoids include furan, methylfuran, furfural, methylfurfural, and furanone as a sum. We select maleic anhydride and furanoids here because they are robust indicators of photochemical oxidation in BB smoke (*14*, *19*). Observations are shown as circles, and MCM_{BBVOC} results are shown as solid lines.

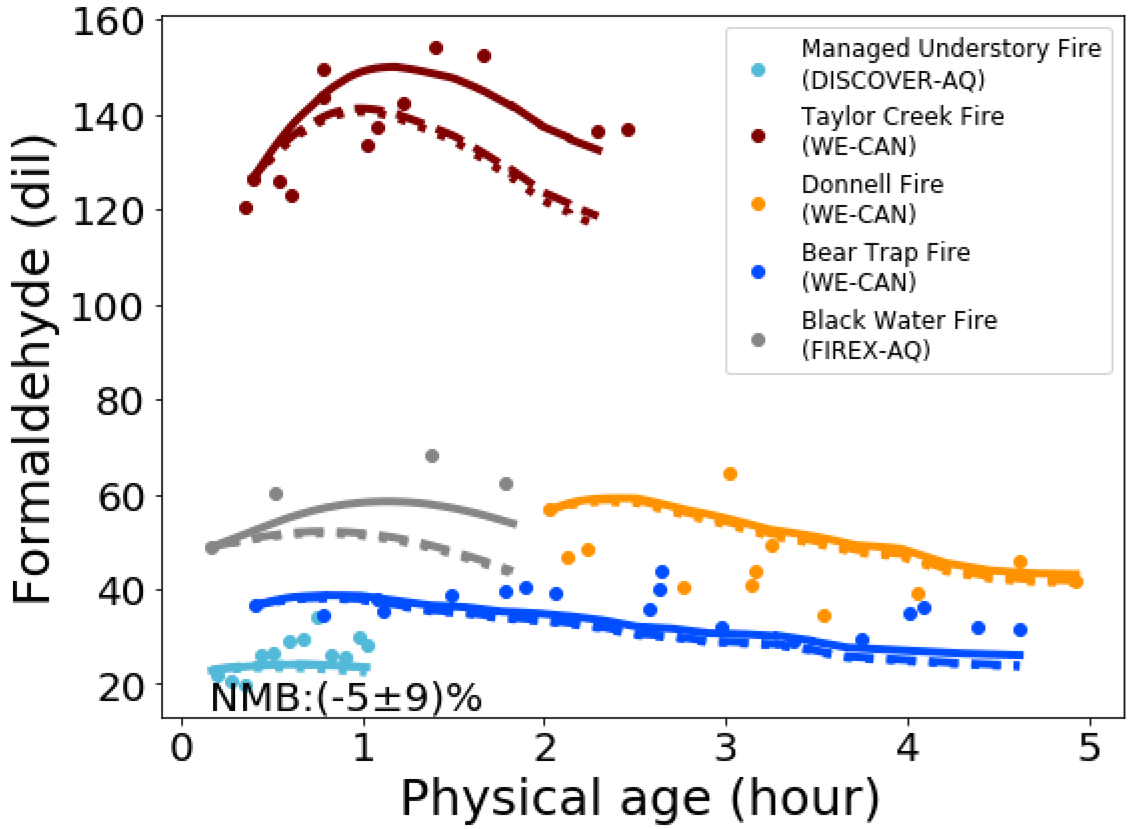

Fig. S3. Evolution of formaldehyde dilution-corrected mixing ratio, colored by different biomass-burning plumes. Observed data are shown as circles, and model simulations from MCM_{BBVOC} (solid line), MCM_{GCVOC} (dashed line), and GEOS-Chem (dotted line) are overlaid for formaldehyde and acetaldehyde. Insets show the normalized mean bias (NMB) between MCM_{BBVOC} and observations; NMB values for MCM_{GCVOC} and GEOS-Chem, available when the corresponding VOCs are included in each mechanism, are summarized in Table S1.

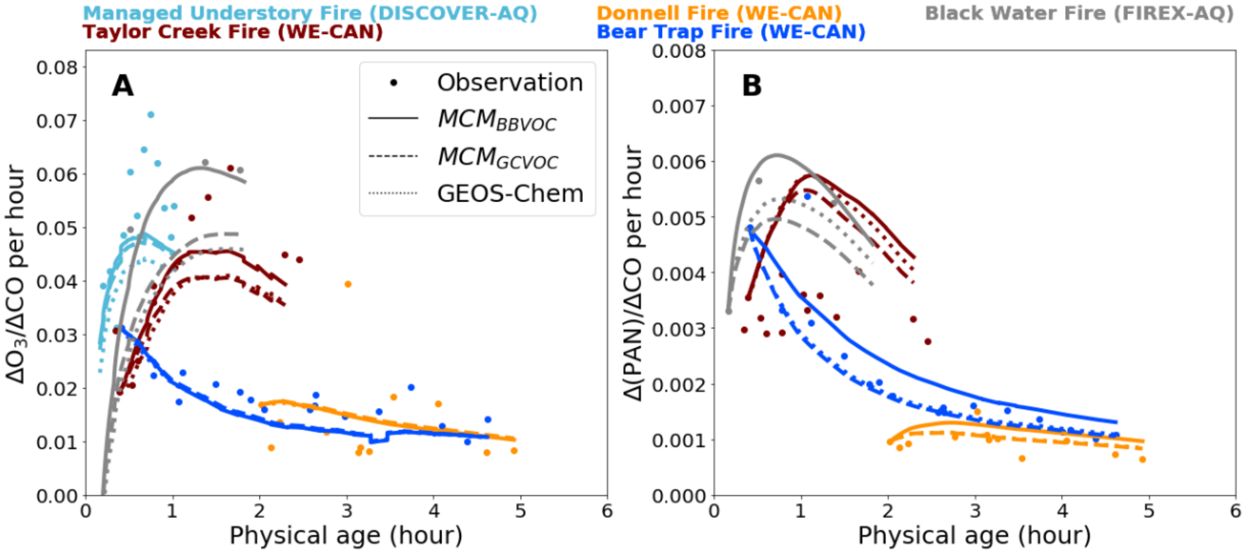

Fig. S4. Evolution of $\Delta O_3/\Delta CO$ and $\Delta PAN/\Delta CO$ per hour within 5 hours of physical aging. (a) $\Delta O_3/\Delta CO$ per hour. (b) $\Delta PAN/\Delta CO$ per hour. Observations are shown in circles, and model results are shown by lines (MCM_{BBVOC} as solid lines; MCM_{GCVOC} dashed lines; GEOS-Chem dotted lines). Both circles and lines are colored by different fires.

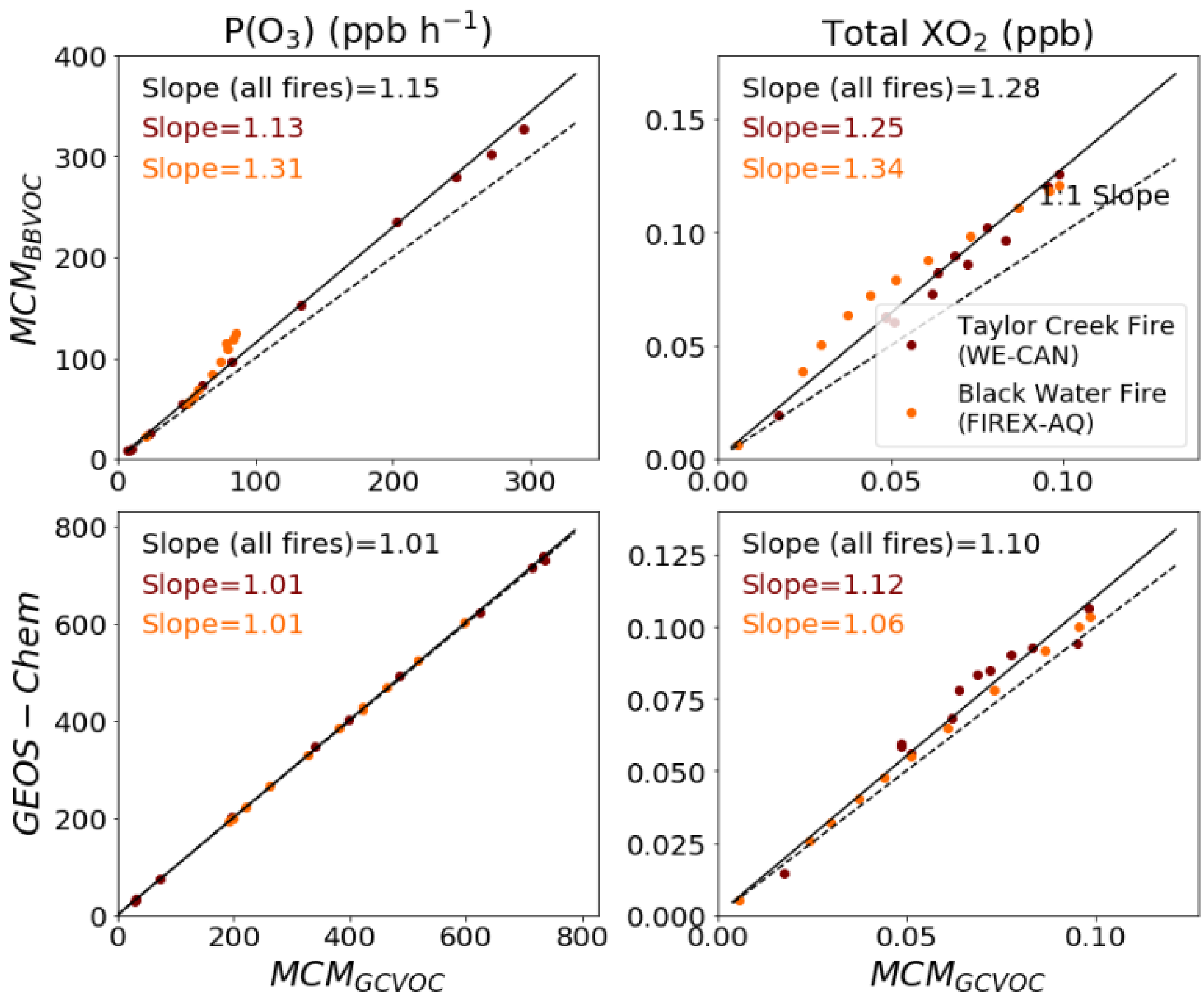

Fig. S5. Model comparison of ozone production rates ($P(O_3)$) and total XO_2 concentrations in the Taylor Creek and Black Water Fires, colored by different fires. The model results are from MCM_{BBVOC}, MCM_{GCVOC}, and GEOS-Chem, which are binned every 10 min. Also shown are the best-fit line of scatters from the two fires (solid) and 1: 1 line (dashed).

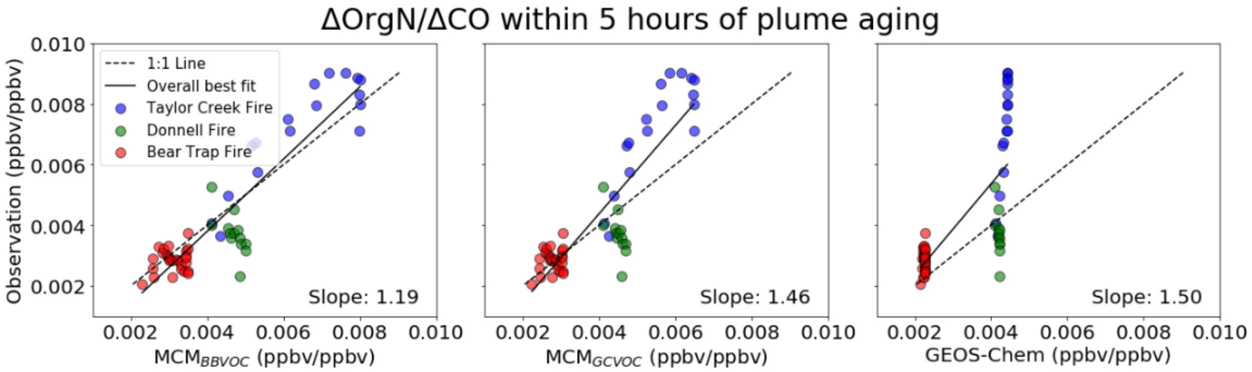

Fig. S6. Comparisons of observed ΔOrgN/ΔCO (ppbv/ppbv) and model results from MCM$_{BBVOC}$, MCM$_{GCVOC}$, and GEOS-Chem, colored by different fires. Model results are interpolated using observational sampling time for visualization. The solid lines represent the best fit between the model and observations, with slopes shown as insets. Also, the 1: 1 line is shown as dashed black lines.

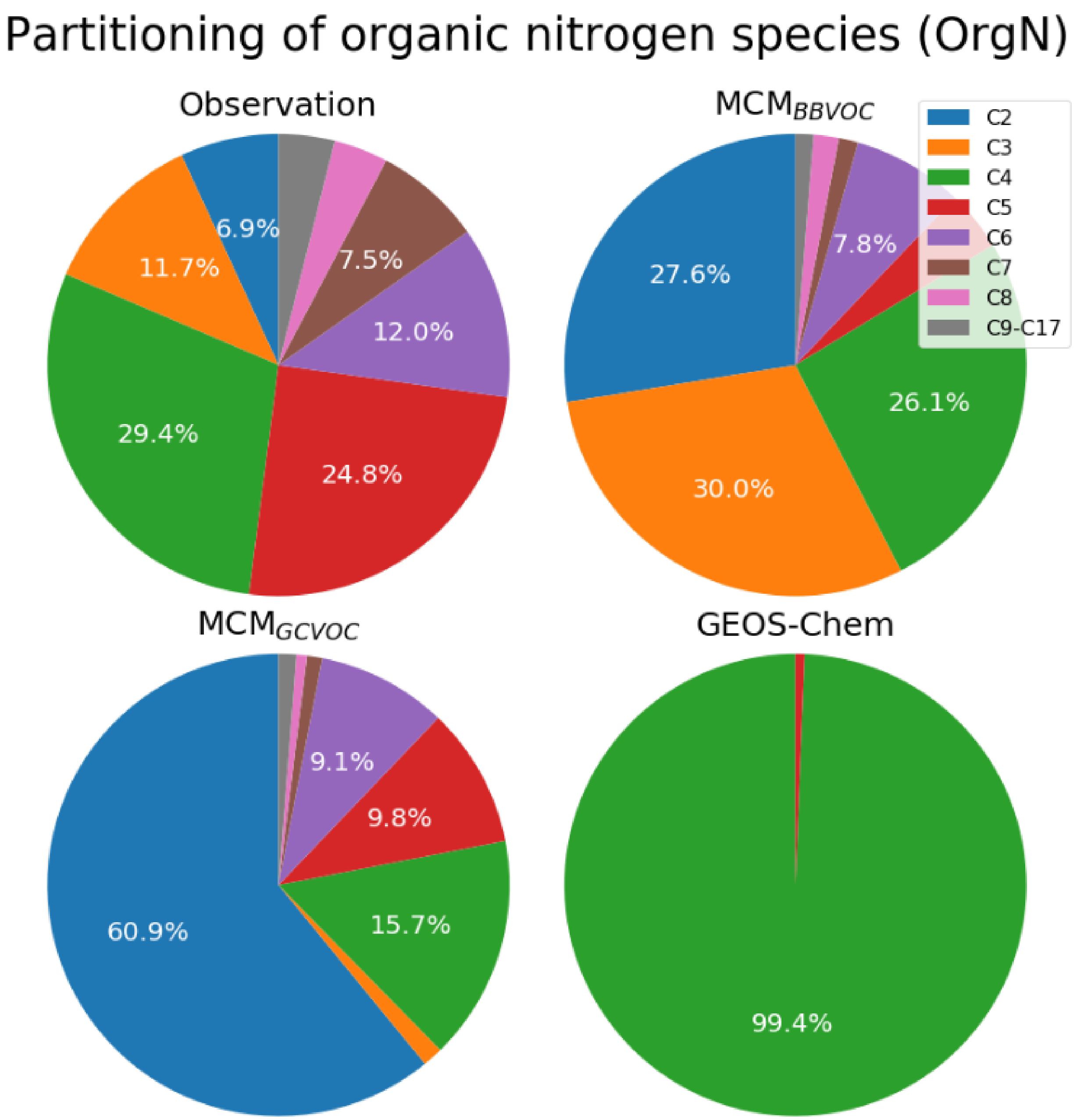

Fig. S7. Observed and modeled fraction of individual OrgN group to the total OrgN in fire plume centers. Results are averaged across three WE-CAN fires in the first 5 hours, with each segment representing the fraction of a different carbon chain length of nitrates to the total OrgN. The plume center is defined as the portion of each plume transect where CO is above the top 5% of the plume. The enhancement of each species is calculated by subtracting a transect-specific background mixing ratio for that species. Note the observed OrgN is highly uncertain.

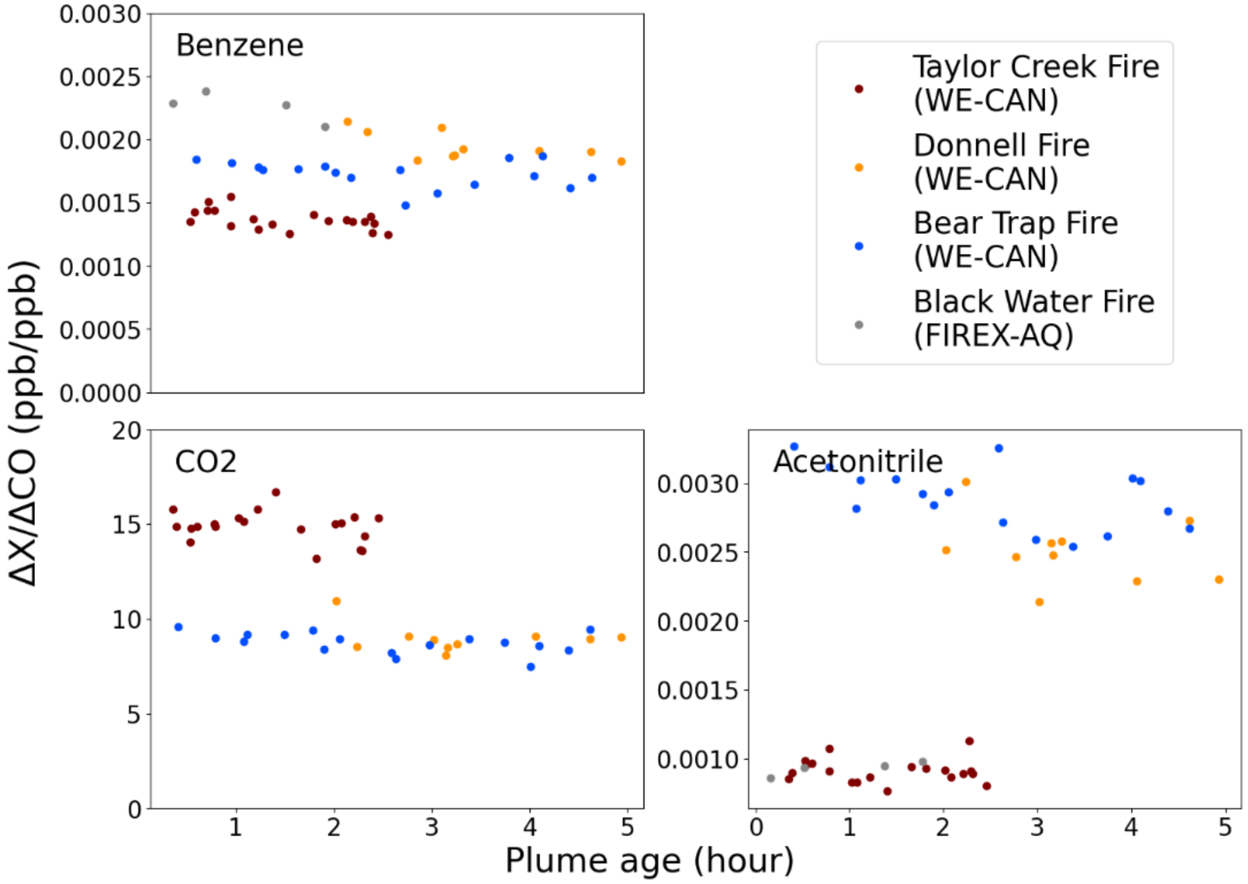

Fig. S8. Evolution of benzene, CO_2, and acetonitrile concentrations against CO within 5 hours of physical aging, colored by different BB plumes. Observations are shown in circles.

Table S1. Average model biases of "short-lived" VOC groups in fresh plumes

VOC name	Measurement uncertainty	MCM_{BBVOC}	MCM_{GCVOC}	GEOS-Chem	VOC type
Furanoids *	±(15–50)%	(-14±38)%	NaN	NaN	Primary
2,5-Dimethylfuran	±50%	(-100±3)%	NaN	NaN	Primary
1,3-Butadiene	±50%	(-35±21)%	NaN	NaN	Primary
Isoprene	±50%	(0±70)%	(0±79)%	(0±81)%	Primary
Monoterpenes *	±50%	(-18±30)%	(-42±35)%	(-49±18)%	Primary
Xylenes *	±15%	(0±1)%	(0±6)%	(-3±15)%	Primary
2-Butenal	±50%	(0±31)%	NaN	NaN	Primary
Cresol	±50%	(-7±50)%	(-24±28)%	-27±39)%	Primary
Guaiacol	±50%	(-71±55)%	NaN	NaN	Primary
Syringol	±50%	(0±74)%	NaN	NaN	Primary
Sesquiterpenes	±50%	(-100±100)%	NaN	NaN	Primary
Phenol	±50%	(-15±8)%	(-25±9)%	(-30±13)%	Primary
Formaldehyde	±40%	(-5±7)%	(-12±5)%	(-13±8)%	Secondary
Acetaldehyde	±15%	(-1±16)%	(-5±14)%	(-7±18)%	Secondary
Maleic anhydride	±50%	(-4±23)%	NaN	NaN	Secondary
3-Methylfuran	±(15–50)%	(-29±68)%	NaN	NaN	Secondary
Lumped C ≥ 3 aldehydes *	±50%	(-0±17)%	(-1±17)%	(-17±8)%	Secondary
Glyoxal	±50%	(0±36)%	(-11±43)%	(-13±46)%	Secondary
Formic acid	±50%	(-46±17)%	(-46±17)%	(-35±8)%	Secondary
MACR + MVK*	±15%	(8±60)%	(-1±46)%	(-2±42)%	Secondary
MEK	±15%	(-0±2)%	(0±0)%	(5±10)%	Secondary
Acetic acid + glycolaldehyde *	±(15–50)%	(31±23)%	(25±15)%	(31±22)%	Secondary
Methylglyoxal	±50%	(-9±11)%	(-17±13)%	(-5±20)%	Secondary
Hydroxy acetone	±50%	(3±13)%	(6±14)%	(1±9)%	Secondary
Phenol	±50%	(-15±8)%	(-25±9)%	(-30±13)%	Secondary
Benzaldehyde	±50%	(17±8)%	(-9±14)%	(-8±15)%	Secondary
Acrolein	±50%	(2±4)%	NaN	NaN	Secondary
2,3-Butanedione	±50%	(-40±79)%	NaN	NaN	Secondary

Note: the 43 VOCs include 22 individual VOCs and another 21 VOCs from 5 lumped VOCs. The 5 lumped VOCs are highlighted by * symbol. For example, furanoids include furan, 2-methylfuran, furfural, 5-methylfurfural, and furanone. Monoterpenes include alpha-pinene and beta-pinene. Xylenes include o-, m-, p-xylenes, and ethylbenzene. Lumped C ≥ 3 aldehydes include propanal, butanal, 2-methylpropanal, 2-methylbutanal, hexanal, and heptanal. MACR+MVK includes methacrolein and methyl vinyl ketone. Acetic acid +glycolaldehyde include glycolaldehyde and acetic acid.

Table S2. Net ozone production rate (% per hour) within the 5 hours of plume aging

Plume age (hours)	Mean ± Standard deviation	min	max	Number of datapoints
0-1	4.2 ± 1.5	1.9	7.1	20
1-2	4.1 ± 1.8	1.7	6.2	13
2-3	2.0 ± 1.2	0.9	4.5	11
3-4	1.4 ± 0.9	0.8	3.9	11
4-5	1.2 ± 0.3	0.8	1.7	7

Table S3. Details of five selected fire plumes simulated in this work

Fire names	Field campaign	Plume age (min)	Burned fuels	State	Fire location	Initial OHR_{VOC} (s^{-1})	Initial OHR_{NOx} (s^{-1})	Initial CO (ppm)	Initial HONO (ppb ppm_{CO}^{-1})	θ value [c]	MCE [d]
Taylor Creek	WE-CAN [a]	21–150	Tanoak, fir, and pine forest	OR	42.52°N, 123.58°W	142.2	15.7	5.0	10	0.111	0.94
Donnell	WE-CAN	120–300	Fir, pine, juniper, and oak forest	CA	38.35°N, 119.93°W	94.5	2.2	3.3	1	0.023	0.90
Bear Trap	WE-CAN	25–300	Fir, pine, juniper, aspen, spruce and oak forest	UT	39.29°N, 109.87°W	86.1	1.5	2.0	1.4	0.018	0.91
Blackwater	FIREX-AQ	10–110	Oak and pine forests, shrub and litter understory	FL	30.98°N, 86.93°W	109.1	12.6	2.1	3	0.115	0.93
Managed Understory	DISCOVER-AQ [b]	0–60	Residual tree logs (pine, oak) and weeds	GA	32.39°N, 82.85°W	54.4	2.8	1.1	1.1	0.051	0.90

[a] We analyzed the freshest smoke sampled using the averaged plume center mixing ratios, defined by the top 5% of CO observations for each transect (*17*, *29*, *40*). [b] The plume was sampled at its origin to derive emission factors and followed about 13.6 km downwind to observe chemical changes during the first hour of atmospheric aging (*32*). [c] θ value is calculated by dividing total OH reactivity from NO_x by that from VOCs. [c] MCE values for each emission transect are taken from previous studies based on plume-integrated excess CO and CO_2 mixing ratios (*11*, *32*, *52*).

Table S4. Summary of used metrics to determine photochemical ozone regime

Metric	NO_x sensitivity	VOC sensitivity	Pros and cons	References
FNR	High FNR<br>e.g., ratio > 2[a]	Low FNR<br>e.g., ratio < 1[a]	Pros: easy to get required data<br>Cons: the threshold vary regionally	(*55*)
FNR vs NO	FNR ratio strongly decreases with small increases in NO	FNR ratio is unresponsive to NO changes	Pros: easy to get required data and do not rely on specific threshold.<br>Cons: assuming CH_2O is the proxy of total VOC abundance	(*57*)
θ value	High θ value,<br>e.g., ratio > 0.2	Low θ value<br>e.g., ratio < 0.01	Pros: comprehensive information<br>Cons: high requirement for detailed VOC measurements	(*58*)
L_{ROx}: L_{NOx} ratio	Low L_{ROx}: L_{NOx}<br>e.g., ratio < 1	High L_{ROx}: L_{NOx}<br>e.g., ratio > 1	Pros: reliable and robust<br>Cons: hard to get observed evaluation	(*55*, *77*)

a. the value used from Duncan et al. (*55*)

b. Because the metric of FNR–NO relationship is not commonly used, we provide the rational here. In NO_x-limited regimes, added NO accelerates RO_2-to-NO_2 formation but has negligible impacts on formaldehyde formation (which is near saturation in VOC-rich environments). Thus, FNR declines sharply with increasing NO. In VOC-limited regimes, abundant NO_x saturates NO_2 production, while formaldehyde remains suppressed by limited VOC oxidation, making FNR relatively unresponsive to NO changes. These dynamics mechanistically distinguish plume regimes: NO_x-limited regimes are characterized by FNR sensitivity to NO, whereas VOC-limited regimes exhibit FNR stability.

Table S5. Net PAN production rate (% per hour) within the 5 hours of plume aging

Plume age (hours)	Mean ± Standard deviation	min	max	Number of datapoints
0-1	0.4 ± 0.1	0.3	0.6	10
1-2	0.3 ± 0.1	0.2	0.5	11
2-3	0.2 ± 0.1	0.1	0.3	11
3-4	0.1 ± 0.0	0.1	0.2	11
4-5	0.1 ± 0.0	0.1	0.1	7

Table S6. Summary of instrumentation for trace gases and radiation used in this work

**DISCOVER-AQ**			
**Species**	**Instrument**	**Accuracy**	**References**
VOCs	PTR-ToF-MS[a]	5–40 %	(*78*)
CO	DACOM[b]	<1 ppb	(*79*)
HONO	OP-FTIR	40%	(*80*)
NO, NO_2, and O~~3~~	Chemiluminescence	5-10%	(*81*)
PAN	n/a	n/a	n/a
jNO_2	jNO2 Filter Radiometer	11%	(*82*)
**WE-CAN**			
**Species**	**Instrument**	**Accuracy**	**References**
VOCs	PTR-ToF-MS[a]	15% for calibrated species<br>50% for uncalibrated species	(*11*)
CO	QCL[c]	1 ppb (2σ)	(*83*)
HONO and organic nitrates	Iodide-adduct CIMS[d]	30-40% for HONO<br>100% for organic nitrates	(*45*, *60*, *84*, *85*)
NO, NO_2, and O_3	Chemiluminescence Detector	2–4%	(*81*)
PAN	CIMS[d]	12 % or 25 ppt	(*86*)
Photolysis Frequencies	HARP – Actinic Flux[e]	Molecule dependent	(*87*)
**FIREX-AQ**			
**Species**	**Instrument**	**Accuracy**	**References**
VOCs	PTR-ToF-MS[a]	15% for calibrated species<br>50% for uncalibrated species	(*52*)
CO	DACOM[b]	2%	(*79*)
HONO	I^- CIMS[d]	30–40%	(*88*)
NO, NO_2, and O_3	Chemiluminescence Detector	2–7%	(*62*, *81*, *89*)
PAN	CIMS[e]	12 % or 25 ppt	(*96*)
Photolysis Frequencies	CAFS[f]	Molecule dependent	(*97*)

[a] Proton-transfer-reaction time-of-flight mass spectrometer. [b] Differential Absorption Carbon Monoxide Measurement. [c]Quantum cascade laser instrument. [d]Iodide-adduct chemical-ionization mass spectrometer. [e]Chemical-ionization mass spectrometer. [e]high-performance instrumented airborne platform for environmental research (HIAPER) Airborne Radiation—Actinic Flux (HARP-Actinic Flux). [f]Charged-coupled device Actinic Flux Spectroradiometer.

Table S7. VOCs that are used to initialize model experiments.

Formula	Name	MCM species	GEOS-Chem species	VOC group	VOC type	GESO-Chem	MCM_{GCVOC}	MCM_{BBVOC}
C2H2*	Ethyne	C2H2	C2H2	Alkyne	P	X	X	X
C2H4*	Ethene	C2H4	C2H4	Alkene	P	X	X	X
C2H6*	Ethane	C2H6	C2H6	Alkane	P	X	X	X
C3H6*	Lumped C≥3 alkenes	-	PRPE	Alkene	P	X	-	-
C3H6*	Propene	C3H6	-	Alkene	P	-	X	X
C4H8*	1-Butene	BUT1ENE	-	Alkene	P	-	X	X
C4H8*	Trans-2-butene	TBUT2ENE	-	Alkene	P	-	X	X
C4H8*	Cis-2-butene	CBUT2ENE	-	Alkene	P	-	X	X
C5H10*	1-Pentene	PENT1ENE	-	Alkene	P	-	X	X
C6H12*	1-Hexene	HEX1ENE	-	Alkene	P	-	X	X
C3H8*	Propane	C3H8	C3H8	Alkane	P	X	X	X
C4H10*	Lumped C≥4 alkanes	-	ALK4	Alkane	P	X	-	-
C4H10*	n-Butane	NC4H10	ALK4	Alkane	P	-	X	X
C4H10*	i-Butane	IC4H10	ALK4	Alkane	P	-	X	X
C5H10*	n-Pentane	NC5H12	ALK4	Alkane	P	-	X	X
C5H10*	i-Pentane	IC5H12	ALK4	Alkane	P	-	X	X
C6H14*	n-Hexane	NC6H14	ALK4	Alkane	P	-	X	X
C6H14*	3-Methylpentane	M3PE	ALK4	Alkane	S	-	X	X
C6H14*	2,2-Dimethylbutane	M22C4	ALK4	Alkane	P	-	X	X
C7H16*	n-Heptane	NC7H16	ALK4	Alkane	P	-	X	X
C8H18*	n-Octane	NC8H18	ALK4	Alkane	P	-	X	X
C9H20*	n-Nonane	NC9H20	ALK4	Alkane	P	-	X	X
C10H22*	n-Decane	NC10H22	ALK4	Alkane	P	-	X	X
C11H24*	Undecane	NC11H24	ALK4	Alkane	P	-	X	X
HCHO	Formaldehyde	HCHO	CH2O	Saturated aliphatic aldehyde	S	X	X	X
CH3CHO	Acetaldehyde	CH3CHO	ALD2	Saturated aliphatic aldehyde	S	X	X	X
C3H6O	Lumped C≥3 aldehydes	-	RCHO	Saturated aliphatic aldehyde	S	X	-	-
C3H6O	Propanal	C2H5CHO	-	Saturated aliphatic aldehyde	S	-	X	X
C4H8O	Butanal	C3H7CHO	-	Saturated aliphatic aldehyde	S	-	X	X
C4H8O	2-Methylpropanal	IPRCHO	-	Saturated aliphatic aldehyde	S	-	X	X
C5H10O	2-Methylbutanal	BUT2CHO	-	Saturated aliphatic aldehyde	S	-	X	X

C6H12O	Hexanal	C5H11CHO	-	Saturated aliphatic aldehyde	S	-	X	X
C7H14O	Heptanal	C6H13CHO	-	Saturated aliphatic aldehyde	S	-	X	X
C2H2O2	Glyoxal	GLYOX	GLYX	Saturated aliphatic aldehyde	S	X	X	X
C6H6	Benzene	BENZENE	BENZ	Benzenoid	P	X	X	X
C7H8	Toluene	TOLUENE	TOLU	Benzenoid	P	X	X	X
C8H10	Xylenes	-	XYLE	Benzenoid	P	X	-	-
C8H10	p-Xylene	PXYL	-	Benzenoid	P	-	X	X
C8H10	m-Xylene	MXYL	-	Benzenoid	P	-	X	X
C8H10	o-Xylene	OXYL	-	Benzenoid	P	-	X	X
C8H10	Ethylbenzene	EBENZ	-	Benzenoid	P	-	X	X
HCOOH	Formic acid	HCOOH	HCOOH	Organic acid	S	X	X	X
C2H4O2	Acetic acid	CH3CO2H	ACTA	Organic acid	S	X	X	X
C5H8	Isoprene	C5H8	ISOP	Biogenic	P	X	X	X
C10H16	Monoterpenes	-	MTPA	Biogenic	P	X	-	-
C10H16	a-Pinene	APINENE	-	Biogenic	P	-	X	X
C10H16	b-Pinene	BPINENE	-	Biogenic	P	-	X	X
C4H6O	Methacrolein	MACR	MACR	Biogenic	S	X	X	X
C4H6O	Methyl vinyl ketone	MVK	MVK	Biogenic	S	X	X	X
CH4O	Methanol	CH3OH	MOH	-	S	X	X	X
C2H6O	Ethanol	C2H5OH	EOH	-	S	X	X	X
C3H6O	Acetone	CH3COCH3	ACET	Ketone	S	X	X	X
C4H8O	Methyl ethyl ketone	MEK	MEK	Ketone	S	X	X	X
C2H4O2	Glycolaldehyde	HOCH2CHO	GLYC	-	S	X	X	X
C2H6S	Dimethyl sulfide	DMS	DMS	-	P	X	X	X
C3H4O2	Methylglyoxal	MGLYOX	MGLY	-	S	X	X	X
C3H6O2	Hydroxyacetone	ACETOL	HAC	Biogenic	S	X	X	X
C6H6O	Phenol	PHENOL	PHEN	Benzenoid	P	X	X	X
C7H6O	Benzaldehyde	BENZAL	BALD	-	S	X	X	X
C7H8O	o-Cresol	CRESOL	CSL	Benzenoid	P	X	X	X
C4H4O	Furan	FURAN		Furanoid	P			X
C5H6O	2-Methylfuran	M2FURAN		Furanoid	P			X
C5H6O	3-Methylfuran	M3F		Furanoid	S			X
C6H8O	2,5-Dimethylfuran	DIMEFURAN		Furanoid	P			X
C5H4O2	2-Furfural	FURFURAL		Furanoid	P			X
C6H6O2	5-Methylfurfural	MEFURFURAL		Furanoid	P			X
C4H4O2	Furanone	BZFUONE		Furanoid	S			X

C3H4O	Acrolein	ACR		-	S			X
C4H2O3	Maleic anhydride	MALANHY		-	S			X
C4H6	1,3-Butadiene	C4H6		-	P			X
C4H6O	2-Butenal	C4ALDB		-	P			X
C4H6O2	2,3-Butanedione	BIACET		-	S			X
C5H10O	2-Pentanone	MPRK		Ketone	P			X
C5H10O	3-Pentanone	DIEK		Ketone	P			X
C6H6O2	Catechol	CATECHOL		-	P			X
C6H12O	Hexanones	CYHEXOL		Ketone	S			X
C7H8O2	Guaiacol	GUAIACOL		-	P			X
C7H14O	Heptanone	HEPT3ONE		Ketone	S			X
C8H8	Styrene	STYRENE		Benzenoid	P			X
C8H8O	Tolualdehyde	OXYLAL		Benzenoid	S			X
C8H10O2	Creosol	MGUAIACOL		Benzenoid	S			X
C8H10O3	Syringol	SYRINGOL		Benzenoid	P			X
C9H12*	1,2,3-Trimethylbenzene	TM123B		Benzenoid	P			X
C9H12*	Isopropylbenzene	IPBENZ		Benzenoid	P			X
C9H12*	3-Ethyltoluene	METHTOL		Benzenoid	P			X
C9H12*	4-Ethyltoluene	PETHTOL		Benzenoid	P			X
C9H12*	1,3,5-Trimethylbenzene	TM135B		Benzenoid	P			X
C9H12*	2-Ethyltoluene	OETHTOL		Benzenoid	P			X
C9H14O4	Pinic acid	PINIC		-	S			X
C10H16O3	Pinonic acid	PINONIC		Biogenic	S			X
C15H24	Sesquiterpene	BCARY		-	P			X

P: Primary VOCs; S: Secondary VOCs;
[a] Formic acid and acetic acid are currently classified as a primary VOC in prevailing mechanisms. However, these mechanisms likely neglect a crucial secondary source for formic acid (*76*).
*VOCs that are initialized by scaling CO with emission ratios from recent emission reports (*11*, *38*).

Table S8. The settings of measured photolysis frequencies

Photolysis	WE-CAN flights	Blackwater Fire	Managed Understory Fire
J(O_3+hv=O_2+O_{1D})	X		
J(NO_2)	X	X	X
J(H_2O_2)	X		
J(NO_3+hv=NO+O_2)	X		
J(NO_3+hv=NO_2+O_{3P})	X		
J(HONO)	X	X	
J(HNO_3)	X	X	
J(CH_2O+hv=H+HCO)	X	X	
J(CH_2O+hv=H_2+CO)	X	X	
J(CH_3CHO)	X		
J(C_2H_5CHO)	X		
J(CH_3OOH)		X	
J(CH_3COCH3)	X		
J(MEK)	X		
J(CHOCHO+hv=2CO+H_2)	X	X	
J(CHOCHO+hv=CH_2O+CO)	X	X	
J(CHOCHO+hv=2HCO)	X	X	
J(CH_3COCHO)	X		
J(CH3COCOCH3)	X		
J(O_3+hv=O_{3P})		X	
J(CH_3CHO)		X	

Note: This table details the specific photolysis frequencies measured in various experimental settings: WE-CAN flights, Blackwater Fire, and Managed Understory Fire. In the WE-CAN flights, actinic flux was measured using the High-Resolution Actinic Flux Spectroradiometer (HARP), and photolysis rates were calculated utilizing the Tropospheric Ultraviolet and Visible (TUV) model. During the Blackwater Fire, similar methods were employed, but the actinic flux was measured using the Charged-coupled device Actinic Flux Spectroradiometers (CAFS). For the Managed Understory Fire, the photolysis frequency of NO_2 (J(NO_2)) was specifically measured using a J(NO_2) Filter Radiometer. The 'X' marks indicate the specific photolysis frequencies measured in each setting.

REFERENCES

1. H. B. Singh, C. Cai, A. Kaduwela, A. Weinheimer, A. Wisthaler, Interactions of fire emissions and urban pollution over California: Ozone formation and air quality simulations. *Atmos. Environ.* **56**, 45–51 (2012).

2. I. Bourgeois, J. Peischl, J. Andrew Neuman, S. S. Brown, C. R. Thompson, K. C. Aikin, H. M. Allen, H. Angot, E. C. Apel, C. B. Baublitz, J. F. Brewer, P. Campuzano-Jost, R. Commane, J. D. Crounse, B. C. Daube, J. P. DiGangi, G. S. Diskin, L. K. Emmons, A. M. Fiore, G. I. Gkatzelis, A. Hills, R. S. Hornbrook, L. Gregory Huey, J. L. Jimenez, M. Kim, F. Lacey, K. McKain, L. T. Murray, B. A. Nault, D. D. Parrish, E. Ray, C. Sweeney, D. Tanner, S. C. Wofsy, T. B. Ryerson, Large contribution of biomass burning emissions to ozone throughout the global remote troposphere. *Proc. Natl. Acad. Sci. U.S.A.* **118**, e2109628118 (2021).

3. L. Zhang, D. J. Jacob, N. V. Downey, D. A. Wood, D. Blewitt, C. C. Carouge, A. van Donkelaar, D. B. A. Jones, L. T. Murray, Y. Wang, Improved estimate of the policy-relevant background ozone in the United States using the GEOS-Chem global model with 1/2° × 2/3° horizontal resolution over North America. *Atmos. Environ.* **45**, 6769–6776 (2011).

4. J. L. Wilkins, G. Pouliot, K. Foley, W. Appel, T. Pierce, The impact of US wildland fires on ozone and particulate matter: A comparison of measurements and CMAQ model predictions from 2008 to 2012. *Int. J. Wildland Fire* **27**, 684–698 (2018).

5. D. A. Jaffe, O. R. Cooper, A. M. Fiore, B. H. Henderson, G. S. Tonnesen, A. G. Russell, D. K. Henze, A. O. Langford, M. Lin, T. Moore, Scientific assessment of background ozone over the U.S.: Implications for air quality management. *Elementa* **6**, 56 (2018).

6. L. Jin, W. Permar, V. Selimovic, D. Ketcherside, R. J. Yokelson, R. S. Hornbrook, E. C. Apel, I.-T. Ku, J. L. Collett Jr., A. P. Sullivan, D. A. Jaffe, J. R. Pierce, A. Fried, M. M. Coggon, G. I. Gkatzelis, C. Warneke, E. V. Fischer, L. Hu, Constraining emissions of volatile organic compounds from western US wildfires with WE-CAN and FIREX-AQ airborne observations. *Atmos. Chem. Phys.* **23**, 5969–5991 (2023).

7. W. Tang, L. K. Emmons, R. R. Buchholz, C. Wiedinmyer, R. H. Schwantes, C. He, R. Kumar, G. G. Pfister, H. M. Worden, R. S. Hornbrook, E. C. Apel, S. Tilmes, B. Gaubert, S.-E.

Martinez-Alonso, F. Lacey, C. D. Holmes, G. S. Diskin, I. Bourgeois, J. Peischl, T. B. Ryerson, J. W. Hair, A. J. Weinheimer, D. D. Montzka, G. S. Tyndall, T. L. Campos, W. Tang, Effects of fire diurnal variation and plume rise on U.S. air quality during FIREX-AQ and WE-CAN based on the Multi-Scale Infrastructure for Chemistry and aerosols (MUSICAv0). *J. Geophys. Res. Atmos.* **127**, e2022JD036650 (2022).

8. L. Xu, J. D. Crounse, K. T. Vasquez, H. Allen, P. O. Wennberg, I. Bourgeois, S. S. Brown, P. Campuzano-Jost, M. M. Coggon, J. H. Crawford, J. P. DiGangi, G. S. Diskin, A. Fried, E. M. Gargulinski, J. B. Gilman, G. I. Gkatzelis, H. Guo, J. W. Hair, S. R. Hall, H. A. Halliday, T. F. Hanisco, R. A. Hannun, C. D. Holmes, L. G. Huey, J. L. Jimenez, A. Lamplugh, Y. R. Lee, J. Liao, J. Lindaas, J. A. Neuman, J. B. Nowak, J. Peischl, D. A. Peterson, F. Piel, D. Richter, P. S. Rickly, M. A. Robinson, A. W. Rollins, T. B. Ryerson, K. Sekimoto, V. Selimovic, T. Shingler, A. J. Soja, J. M. S. Clair, D. J. Tanner, K. Ullmann, P. R. Veres, J. Walega, C. Warneke, R. A. Washenfelder, P. Weibring, A. Wisthaler, G. M. Wolfe, C. C. Womack, R. J. Yokelson, Ozone chemistry in western U.S. wildfire plumes. *Sci. Adv.* **7**, 3648 (2021).

9. T. S. Carter, C. L. Heald, J. H. Kroll, E. C. Apel, D. Blake, M. Coggon, A. Edtbauer, G. Gkatzelis, R. S. Hornbrook, J. Peischl, E. Y. Pfannerstill, F. Piel, N. G. Reijrink, A. Ringsdorf, C. Warneke, J. Williams, A. Wisthaler, L. Xu, An improved representation of fire non-methane organic gases (NMOGs) in models: Emissions to reactivity. *Atmos. Chem. Phys.* **22**, 12093–12111 (2022).

10. C. E. Stockwell, R. J. Yokelson, S. M. Kreidenweis, A. L. Robinson, P. J. Demott, R. C. Sullivan, J. Reardon, K. C. Ryan, D. W. T. Griffith, L. Stevens, Trace gas emissions from combustion of peat, crop residue, domestic biofuels, grasses, and other fuels: Configuration and Fourier transform infrared (FTIR) component of the fourth Fire Lab at Missoula Experiment (FLAME-4). *Atmos. Chem. Phys.* **14**, 9727–9754 (2014).

11. W. Permar, Q. Wang, V. Selimovic, C. Wielgasz, R. J. Yokelson, R. S. Hornbrook, A. J. Hills, E. C. Apel, I. Ku, Y. Zhou, B. C. Sive, A. P. Sullivan, J. L. Collett, T. L. Campos, B. B. Palm, Q. Peng, J. A. Thornton, L. A. Garofalo, D. K. Farmer, S. M. Kreidenweis, E. J. T. Levin, P. J. DeMott, F. Flocke, E. V. Fischer, L. Hu, Emissions of trace organic gases from western U.S. wildfires based on WE-CAN aircraft measurements. *J. Geophys. Res. Atmos.* **126**, e2020JD033838 (2021).

12. A. R. Koss, K. Sekimoto, J. B. Gilman, V. Selimovic, M. M. Coggon, K. J. Zarzana, B. Yuan, B. M. Lerner, S. S. Brown, J. L. Jimenez, J. Krechmer, J. M. Roberts, C. Warneke, R. J. Yokelson, J. de Gouw, Non-methane organic gas emissions from biomass burning: Identification, quantification, and emission factors from PTR-ToF during the FIREX 2016 laboratory experiment. *Atmos. Chem. Phys.* **18**, 3299–3319 (2018).

13. J. Jiang, W. P. L. Carter, D. R. Cocker, K. C. Barsanti, Development and evaluation of a detailed mechanism for gas-phase atmospheric reactions of furans. *ACS Earth Space Chem.* **4**, 1254–1268 (2020).

14. M. M. Coggon, C. Y. Lim, A. R. Koss, K. Sekimoto, B. Yuan, J. B. Gilman, D. H. Hagan, V. Selimovic, K. J. Zarzana, S. S. Brown, J. M. Roberts, M. Müller, R. Yokelson, A. Wisthaler, J. E. Krechmer, J. L. Jimenez, C. Cappa, J. H. Kroll, J. De Gouw, C. Warneke, OH chemistry of non-methane organic gases (NMOGs) emitted from laboratory and ambient biomass burning smoke: Evaluating the influence of furans and oxygenated aromatics on ozone and secondary NMOG formation. *Atmos. Chem. Phys.* **19**, 14875–14899 (2019).

15. Z. Lu, J. Wang, X. Xu, X. Chen, S. Kondragunta, O. Torres, E. M. Wilcox, J. Zeng, Hourly mapping of the layer height of thick smoke plumes over the Western U.S. in 2020 severe fire season. *Front. Remote Sens.* **2**, doi.org/10.3389/frsen.2021.766628 (2021).

16. J. Wang, S. Roudini, E. J. Hyer, X. Xu, M. Zhou, L. C. Garcia, J. S. Reid, D. A. Peterson, A. M. da Silva, Detecting nighttime fire combustion phase by hybrid application of visible and infrared radiation from Suomi NPP VIIRS. *Remote Sens. Environ.* **237**, 111466 (2020).

17. W. Permar, L. Jin, Q. Peng, K. O'Dell, E. Lill, V. Selimovic, R. J. Yokelson, R. S. Hornbrook, A. J. Hills, E. C. Apel, I.-T. Ku, Y. Zhou, B. C. Sive, A. P. Sullivan, J. L. Collett, B. B. Palm, J. A. Thornton, F. Flocke, E. V. Fischer, L. Hu, Atmospheric OH reactivity in the western United States determined from comprehensive gas-phase measurements during WE-CAN. *Environ. Sci.: Atmos.* **3**, 97 (2023).

18. J. Liao, G. M. Wolfe, R. A. Hannun, J. M. S. Clair, T. F. Hanisco, J. B. Gilman, A. Lamplugh, V. Selimovic, G. S. Diskin, J. B. Nowak, H. S. Halliday, J. P. Digangi, S. R. Hall, K. Ullmann, C. D. Holmes, D. Richter, P. Weibring, E. C. Apel, R. S. Hornbrook, S. S. Brown, J.

A. Neuman, Formaldehyde evolution in US wildfire plumes during the Fire Influence on Regional to Global Environments and Air Quality experiment (FIREX-AQ). *ACP* **5**, 18319–18331 (2021).

19. S. Wang, M. M. Coggon, G. I. Gkatzelis, C. Warneke, I. Bourgeois, T. Ryerson, J. Peischl, P. R. Veres, J. A. Neuman, J. Hair, T. Shingler, M. Fenn, G. Diskin, L. G. Huey, Y. R. Lee, E. C. Apel, R. S. Hornbrook, A. J. Hills, S. R. Hall, K. Ullmann, M. M. Bela, M. K. Trainer, R. Kumar, J. J. Orlando, F. M. Flocke, L. K. Emmons, Chemical tomography in a fresh wildland fire plume: A large eddy simulation (LES) study. *J. Geophys. Res. Atmos.* **126**, e2021JD035203 (2021).

20. I. Bey, D. J. Jacob, R. M. Yantosca, J. A. Logan, B. D. Field, A. M. Fiore, Q. Li, H. Y. Liu, L. J. Mickley, M. G. Schultz, Global modeling of tropospheric chemistry with assimilated meteorology: Model description and evaluation. *J. Geophys. Res. Atmos.* **106**, 23073–23095 (2001).

21. W. P. L. Carter, G. Heo, Development of revised SAPRC aromatics mechanisms. *Atmos. Environ.* **77**, 404–414 (2013).

22. L. K. Emmons, R. H. Schwantes, J. J. Orlando, G. Tyndall, D. Kinnison, J. F. Lamarque, D. Marsh, M. J. Mills, S. Tilmes, C. Bardeen, R. R. Buchholz, A. Conley, A. Gettelman, R. Garcia, I. Simpson, D. R. Blake, S. Meinardi, G. Pétron, The chemistry mechanism in the Community Earth System Model version 2 (CESM2). *J. Adv. Model. Earth Syst.* **12**, e2019MS001882 (2020).

23. S. M. Saunders, M. E. Jenkin, R. G. Derwent, M. J. Pilling, Protocol for the development of the Master Chemical Mechanism, MCM v3 (part A): Tropospheric degradation of non-aromatic volatile organic compounds. *Atmos. Chem. Phys.* **3**, 161–180 (2003).

24. C. Bloss, V. Wagner, M. E. Jenkin, R. Volkamer, W. J. Bloss, J. D. Lee, D. E. Heard, K. Wirtz, M. Martin-Reviejo, G. Rea, J. C. Wenger, M. J. Pilling, Development of a detailed chemical mechanism (MCMv3.1) for the atmospheric oxidation of aromatic hydrocarbons. *Atmos.Chem. Phys.* **5**, 641–664 (2005).

25. M. E. Jenkin, S. M. Saunders, V. Wagner, M. J. Pilling, Protocol for the development of the Master Chemical Mechanism, MCM v3 (part B): Tropospheric degradation of aromatic volatile organic compounds. *Atmos. Chem. Phys.* **3**, 181–193 (2003).

26. M. E. Jenkin, K. P. Wyche, C. J. Evans, T. Carr, P. S. Monks, M. R. Alfarra, M. H. Barley, G. B. McFiggans, J. C. Young, A. R. Rickard, Development and chamber evaluation of the MCM v3.2 degradation scheme for β-caryophyllene. *Atmos.Chem. Phys.* **12**, 5275–5308 (2012).

27. M. E. Jenkin, J. C. Young, A. R. Rickard, The MCM v3.3.1 degradation scheme for isoprene. *Atmos.Chem. Phys.* **15**, 11433–11459 (2015).

28. A. J. Lindsay, D. C. Anderson, R. A. Wernis, Y. Liang, A. H. Goldstein, S. C. Herndon, J. R. Roscioli, C. Dyroff, E. C. Fortner, P. L. Croteau, F. Majluf, J. E. Krechmer, T. I. Yacovitch, W. B. Knighton, E. C. Wood, Ground-based investigation of HO*x* and ozone chemistry in biomass burning plumes in rural Idaho. *Atmos. Chem. Phys.* **22**, 4909–4928 (2022).

29. Q. Peng, B. B. Palm, C. D. Fredrickson, B. H. Lee, S. R. Hall, K. Ullmann, T. Campos, A. J. Weinheimer, E. C. Apel, F. Flocke, W. Permar, L. Hu, L. A. Garofalo, M. A. Pothier, D. K. Farmer, I. T. Ku, A. P. Sullivan, J. L. Collett, E. Fischer, J. A. Thornton, Observations and modeling of NO*x* photochemistry and fate in fresh wildfire plumes. *ACS Earth Space Chem.* **5**, 2652–2667 (2021).

30. M. Ninneman, D. A. Jaffe, The impact of wildfire smoke on ozone production in an urban area: Insights from field observations and photochemical box modeling. *Atmos. Environ.* **267**, 118764 (2021).

31. G. M. Wolfe, T. F. Hanisco, H. L. Arkinson, D. R. Blake, A. Wisthaler, T. Mikoviny, T. B. Ryerson, I. Pollack, J. Peischl, P. O. Wennberg, J. D. Crounse, J. M. St. Clair, A. Teng, L. G. Huey, X. Liu, A. Fried, P. Weibring, D. Richter, J. Walega, S. R. Hall, K. Ullmann, J. L. Jimenez, P. Campuzano-Jost, T. P. Bui, G. Diskin, J. R. Podolske, G. Sachse, R. C. Cohen, Photochemical evolution of the 2013 California Rim Fire: Synergistic impacts of reactive hydrocarbons and enhanced oxidants. *Atmos. Chem. Phys.* **22**, 4253–4275 (2022).

32. M. Müller, B. E. Anderson, A. J. Beyersdorf, J. H. Crawford, G. S. Diskin, P. Eichler, A. Fried, F. N. Keutsch, T. Mikoviny, K. L. Thornhill, J. G. Walega, A. J. Weinheimer, M. Yang, R. J. Yokelson, A. Wisthaler, In situ measurements and modeling of reactive trace gases in a small biomass burning plume. *Atmos. Chem. Phys.* **16**, 3813–3824 (2016).

33. S. K. Akagi, R. J. Yokelson, I. R. Burling, S. Meinardi, I. Simpson, D. R. Blake, G. R. McMeeking, A. Sullivan, T. Lee, S. Kreidenweis, S. Urbanski, J. Reardon, D. W. T. Griffith, T. J. Johnson, D. R. Weise, Measurements of reactive trace gases and variable O3 formation rates in some South Carolina biomass burning plumes. *Atmos. Chem. Phys.* **13**, 1141–1165 (2013).

34. C. Cai, S. Kulkarni, Z. Zhao, A. P. Kaduwela, J. C. Avise, J. A. DaMassa, H. B. Singh, A. J. Weinheimer, R. C. Cohen, G. S. Diskin, P. Wennberg, J. E. Dibb, G. Huey, A. Wisthaler, J. L. Jimenez, M. J. Cubison, Simulating reactive nitrogen, carbon mono*x*ide, and ozone in California during ARCTAS-CARB 2008 with high wildfire activity. *Atmos. Environ.* **128**, 28–44 (2016).

35. V. Selimovic, R. J. Yokelson, G. R. McMeeking, S. Coefield, Aerosol mass and optical properties, smoke influence on O_3, and high NO_3 production rates in a Western U.S. city impacted by wildfires. *J. Geophys. Res. Atmos.* **125**, e2020JD032791 (2020).

36. J. M. Roberts, F. C. Fehsenfeld, S. C. Liu, M. J. Bollinger, C. Hahn, D. L. Albritton, R. E. Sievers, Measurements of aromatic hydrocarbon ratios and NO*x* concentrations in the rural troposphere: Observation of air mass photochemical aging and NO*x* removal. *Atmos. Environ.* **18**, 2421–2432 (1984).

37. J. A. de Gouw, A. M. Middlebrook, C. Warneke, P. D. Goldan, W. C. Kuster, J. M. Roberts, F. C. Fehsenfeld, D. R. Worsnop, M. R. Canagaratna, A. A. P. Pszenny, W. C. Keene, M. Marchewka, S. B. Bertman, T. S. Bates, Budget of organic carbon in a polluted atmosphere: Results from the New England Air Quality Study in 2002. *J. Geophys. Res. Atmos.* **110**, doi.org/10.1029/2004JD005623 (2005).

38. M. O. Andreae, Emission of trace gases and aerosols from biomass burning - An updated assessment. *Atmos. Chem. Phys.* **19**, 8523–8546 (2019).

39. M. R. McGillen, W. P. L. Carter, A. Mellouki, J. J. Orlando, Database for the kinetics of the gas-phase atmospheric reactions of organic compounds. *Earth Syst. Sci. Data* **12**, 1203–1216 (2020).

40. B. B. Palm, Q. Peng, S. R. Hall, K. Ullmann, T. L. Campos, A. Weinheimer, D. Montzka, G. Tyndall, W. Permar, L. Hu, F. Flocke, E. V. Fischer, J. A. Thornton, Spatially resolved photochemistry impacts emissions estimates in fresh wildfire plumes. *Geophys. Res. Lett.* **48**, e2021GL095443 (2021).

41. P. V. Hobbs, P. Sinha, R. J. Yokelson, T. J. Christian, D. R. Blake, S. Gao, T. W. Kirchstetter, T. Novakov, P. Pilewskie, Evolution of gases and particles from a savanna fire in South Africa. *J. Geophys. Res. Atmos.* **108**, 8485 (2003).

42. A. Akherati, Y. He, L. A. Garofalo, A. L. Hodshire, D. K. Farmer, S. M. Kreidenweis, W. Permar, L. Hu, E. V. Fischer, C. N. Jen, A. H. Goldstein, E. J. T. Levin, P. J. DeMott, T. L. Campos, F. Flocke, J. M. Reeves, D. W. Toohey, J. R. Pierce, S. H. Jathar, Dilution and photooxidation driven processes explain the evolution of organic aerosol in wildfire plumes. *Environ. Sci. Atmos.* **2**, 1000–1022 (2022).

43. R. J. Yokelson, J. D. Crounse, P. F. DeCarlo, T. Karl, S. Urbanski, E. Atlas, T. Campos, Y. Shinozuka, V. Kapustin, A. D. Clarke, A. Weinheimer, D. J. Knapp, D. D. Montzka, J. Holloway, P. Weibring, F. Flocke, W. Zheng, D. Toohey, P. O. Wennberg, C. Wiedinmyer, L. Mauldin, A. Fried, D. Richter, J. Walega, J. L. Jimenez, K. Adachi, P. R. Buseck, S. R. Hall, R. Shetter, Emissions from biomass burning in the Yucatan. *Atmos. Chem. Phys.* **9**, 5785–5812 (2009).

44. S. K. Akagi, J. S. Craven, J. W. Taylor, G. R. McMeeking, R. J. Yokelson, I. R. Burling, S. P. Urbanski, C. E. Wold, J. H. Seinfeld, H. Coe, M. J. Alvarado, D. R. Weise, Evolution of trace gases and particles emitted by a chaparral fire in California. *Atmos. Chem. Phys.* **12**, 1397–1421 (2012).

45. Q. Peng, B. B. Palm, K. E. Melander, B. H. Lee, S. R. Hall, K. Ullmann, T. Campos, A. J. Weinheimer, E. C. Apel, R. S. Hornbrook, A. J. Hills, D. D. Montzka, F. Flocke, L. Hu, W. Permar, C. Wielgasz, J. Lindaas, I. B. Pollack, E. V. Fischer, T. H. Bertram, J. A. Thornton,

HONO emissions from Western U.S. wildfires provide dominant radical source in fresh wildfire smoke. *Environ. Sci. Technol.* **54**, 5954–5963 (2020).

46. C. L. Heald, J. De Gouw, A. H. Goldstein, A. B. Guenther, P. L. Hayes, W. Hu, G. Isaacman-Vanwertz, J. L. Jimenez, F. N. Keutsch, A. R. Koss, P. K. Misztal, B. Rappenglück, J. M. Roberts, P. S. Stevens, R. A. Washenfelder, C. Warneke, C. J. Young, Contrasting reactive organic carbon observations in the Southeast United States (SOAS) and Southern California (CalNex). *Environ. Sci. Technol.* **54**, 14923–14935 (2020).

47. M. Ninneman, S. Lyman, L. Hu, E. Cope, D. Ketcherside, D. Jaffe, Investigation of ozone formation chemistry during the Salt Lake Regional Smoke, Ozone, and Aerosol study (SAMOZA). *ACS Earth Space Chem.* **7**, 2521–2534 (2023).

48. N. Theys, R. Volkamer, J. F. Müller, K. J. Zarzana, N. Kille, L. Clarisse, I. De Smedt, C. Lerot, H. Finkenzeller, F. Hendrick, T. K. Koenig, C. F. Lee, C. Knote, H. Yu, M. Van Roozendael, Global nitrous acid emissions and levels of regional oxidants enhanced by wildfires. *Nat. Geosci.* **13**, 681–686 (2020).

49. Q. Peng, B. B. Palm, C. D. Fredrickson, B. H. Lee, S. R. Hall, K. Ullmann, A. J. Weinheimer, E. Levin, P. DeMott, L. A. Garofalo, M. A. Pothier, D. K. Farmer, E. V. Fischer, J. A. Thornton, Direct constraints on secondary HONO production in aged wildfire smoke from airborne measurements over the Western US. *Geophys. Res. Lett.* **49**, e2022GL098704 (2022).

50. X. Liu, Y. Zhang, L. G. Huey, R. J. Yokelson, Y. Wang, J. L. Jimenez, P. Campuzano-Jost, A. J. Beyersdorf, D. R. Blake, Y. Choi, J. M. St, J. D. Clair, D. A. Crounse, G. S. Day, A. Diskin, S. R. Fried, T. F. Hall, L. E. Hanisco, S. King, T. Meinardi, B. B. Mikoviny, J. Palm, A. E. Peischl, I. B. Perring, T. B. Pollack, G. Ryerson, J. P. Sachse, I. J. Schwarz, D. J. Simpson, K. L. Tanner, K. Thornhil, R. J. Ullmann, P. O. Weber, A. Wennberg, G. M. Wisthaler, L. D. Wolfe, Agricultural fires in the southeastern US during SEAC(4)RS: Emissions of trace gases and particles and evolution of ozone, reactive nitrogen, and organic aerosol. *J. Geophys. Res.* **121**, 7383–7414 (2016).

51. K. R. Travis, J. H. Crawford, A. J. Soja, E. M. Gargulinski, R. H. Moore, E. B. Wiggins, G. S. Diskin, J. P. DiGangi, J. B. Nowak, H. Halliday, R. J. Yokelson, J. L. McCarty, I. J. Simpson, D. R. Blake, S. Meinardi, R. S. Hornbrook, E. C. Apel, A. J. Hills, C. Warneke, M. M. Coggon, A. W. Rollins, J. B. Gilman, C. C. Womack, M. A. Robinson, J. M. Katich, J. Peischl, G. I. Gkatzelis, I. Bourgeois, P. S. Rickly, A. Lamplugh, J. E. Dibb, J. L. Jimenez, P. Campuzano-Jost, D. A. Day, H. Guo, D. Pagonis, P. O. Wennberg, J. D. Crounse, L. Xu, T. F. Hanisco, G. M. Wolfe, J. Liao, J. M. St, B. A. Clair, A. Nault, A. E. Fried, A. E. Perring, Emission factors for crop residue and prescribed fires in the Eastern US during FIREX-AQ. *J. Geophys. Res. Atmos.* **128**, e2023JD039309 (2023).

52. G. I. Gkatzelis, M. M. Coggon, C. E. Stockwell, R. S. Hornbrook, H. Allen, E. C. Apel, M. M. Bela, D. R. Blake, I. Bourgeois, S. S. Brown, P. Campuzano-Jost, J. M. St. Clair, J. H. Crawford, J. D. Crounse, D. A. Day, J. P. DiGangi, G. S. Diskin, A. Fried, J. B. Gilman, H. Guo, J. W. Hair, H. S. Halliday, T. F. Hanisco, R. Hannun, A. Hills, L. G. Huey, J. L. Jimenez, J. M. Katich, A. Lamplugh, Y. R. Lee, J. Liao, J. Lindaas, S. A. McKeen, T. Mikoviny, B. A. Nault, J. A. Neuman, J. B. Nowak, D. Pagonis, J. Peischl, A. E. Perring, F. Piel, P. S. Rickly, M. A. Robinson, A. W. Rollins, T. B. Ryerson, M. K. Schueneman, R. H. Schwantes, J. P. Schwarz, K. Sekimoto, V. Selimovic, T. Shingler, D. J. Tanner, L. Tomsche, K. T. Vasquez, P. R. Veres, R. Washenfelder, P. Weibring, P. O. Wennberg, A. Wisthaler, G. M. Wolfe, C. C. Womack, L. Xu, K. Ball, R. J. Yokelson, C. Warneke, Parameterizations of US wildfire and prescribed fire emission ratios and emission factors based on FIREX-AQ aircraft measurements. *Atmos. Chem. Phys.* **24**, 929–956 (2024).

53. H.-A. Kwon, R. J. Park, Y. J. Oak, C. R. Nowlan, S. J. Janz, M. G. Kowalewski, A. Fried, J. Walega, K. H. Bates, J. Choi, D. R. Blake, A. Wisthaler, J.-H. Woo, Top-down estimates of anthropogenic VOC emissions in South Korea using formaldehyde vertical column densities from aircraft during the KORUS-AQ campaign. *Elementa* **9**, 00109 (2021).

54. R. J. Yokelson, M. O. Andreae, S. K. Akagi, Pitfalls with the use of enhancement ratios or normalized excess mixing ratios measured in plumes to characterize pollution sources and aging. *Atmos. Meas. Tech.* **6**, 2155–2158 (2013).

55. B. N. Duncan, Y. Yoshida, J. R. Olson, S. Sillman, R. V. Martin, L. Lamsal, Y. Hu, K. E. Pickering, C. Retscher, D. J. Allen, J. H. Crawford, Application of OMI observations to a space-based indicator of NO*x* and VOC controls on surface ozone formation. *Atmos. Environ.* **44**, 2213–2223 (2010).

56. X. Jin, A. Fiore, A. Fiore, K. F. Boersma, I. De Smedt, L. Valin, Inferring changes in summertime surface ozone-NO*x*-VOC chemistry over U.S. urban areas from two decades of satellite and ground-based observations. *Environ. Sci. Technol.* **54**, 6518–6529 (2020).

57. C. M. Nussbaumer, H. Fischer, J. Lelieveld, A. Pozzer, What controls ozone sensitivity in the upper tropical troposphere? *Atmos. Chem. Phys* **23**, 12651–12669 (2023).

58. F. Kirchner, F. Jeanneret, A. Clappier, B. Krüger, H. Van Den Bergh, B. Calpini, Total VOC reactivity in the planetary boundary layer: 2. A new indicator for determining the sensitivity of the ozone production to VOC and NO*x*. *J. Geophys. Res. Atmos.* **106**, 3095–3110 (2001).

59. M. A. Robinson, Z. C. J. Decker, K. C. Barsanti, M. M. Coggon, F. M. Flocke, A. Franchin, C. D. Fredrickson, J. B. Gilman, G. I. Gkatzelis, C. D. Holmes, A. Lamplugh, A. Lavi, A. M. Middlebrook, D. M. Montzka, B. B. Palm, J. Peischl, B. Pierce, R. H. Schwantes, K. Sekimoto, V. Selimovic, G. S. Tyndall, J. A. Thornton, P. Van Rooy, C. Warneke, A. J. Weinheimer, S. S. Brown, Variability and time of day dependence of ozone photochemistry in western wildfire plumes. *Environ. Sci. Technol.* **55**, 10280–10290 (2021).

60. J. F. Juncosa Calahorrano, J. Lindaas, K. O'Dell, B. B. Palm, Q. Peng, F. Flocke, I. B. Pollack, L. A. Garofalo, D. K. Farmer, J. R. Pierce, J. L. Collett, A. Weinheimer, T. Campos, R. S. Hornbrook, S. R. Hall, K. Ullmann, M. A. Pothier, E. C. Apel, W. Permar, L. Hu, A. J. Hills, D. Montzka, G. Tyndall, J. A. Thornton, E. V. Fischer, Daytime oxidized reactive nitrogen partitioning in Western U.S. wildfire smoke plumes. *J. Geophys. Res. Atmos.* **126**, e2020JD033484 (2021).

61. M. J. Alvarado, C. R. Lonsdale, R. J. Yokelson, S. K. Akagi, H. Coe, J. S. Craven, E. V. Fischer, G. R. McMeeking, J. H. Seinfeld, T. Soni, J. W. Taylor, D. R. Weise, C. E. Wold, Investigating the links between ozone and organic aerosol chemistry in a biomass burning

plume from a prescribed fire in California chaparral. *Atmos. Chem. Phys.* **15**, 6667–6688 (2015).

62. I. Bourgeois, J. Peischl, J. A. Neuman, S. S. Brown, H. M. Allen, P. Campuzano-Jost, M. M. Coggon, J. P. DiGangi, G. S. Diskin, J. B. Gilman, G. I. Gkatzelis, H. Guo, H. A. Halliday, T. F. Hanisco, C. D. Holmes, L. G. Huey, J. L. Jimenez, A. D. Lamplugh, Y. R. Lee, J. Lindaas, R. H. Moore, B. A. Nault, J. B. Nowak, D. Pagonis, P. S. Rickly, M. A. Robinson, A. W. Rollins, V. Selimovic, J. M. St. Clair, D. Tanner, K. T. Vasquez, P. R. Veres, C. Warneke, P. O. Wennberg, R. A. Washenfelder, E. B. Wiggins, C. C. Womack, L. Xu, K. J. Zarzana, T. B. Ryerson, Comparison of airborne measurements of NO, NO_2, HONO, NO_y, and CO during FIREX-AQ. *Atmos. Meas. Tech.* **15**, 4901–4930 (2022).

63. J. Lindaas, I. B. Pollack, L. A. Garofalo, M. A. Pothier, D. K. Farmer, S. M. Kreidenweis, T. L. Campos, F. Flocke, A. J. Weinheimer, D. D. Montzka, G. S. Tyndall, B. B. Palm, Q. Peng, J. A. Thornton, W. Permar, C. Wielgasz, L. Hu, R. D. Ottmar, J. C. Restaino, A. T. Hudak, I. T. Ku, Y. Zhou, B. C. Sive, A. Sullivan, J. L. Collett, E. V. Fischer, Emissions of reactive nitrogen From Western U.S. wildfires during summer 2018. *J. Geophys. Res. Atmos.* **126**, e2020JD032657 (2021).

64. G. M. Wolfe, M. R. Marvin, S. J. Roberts, K. R. Travis, J. Liao, The framework for 0-D atmospheric modeling (F0AM) v3.1. *Geosci. Model Dev.* **9**, 3309–3319 (2016).

65. Z. C. J. Decker, M. A. Robinson, K. C. Barsanti, I. Bourgeois, M. M. Coggon, J. P. Digangi, G. S. Diskin, F. M. Flocke, A. Franchin, C. D. Fredrickson, G. I. Gkatzelis, S. R. Hall, H. Halliday, C. D. Holmes, L. G. Huey, Y. R. Lee, J. Lindaas, A. M. Middlebrook, D. D. Montzka, R. Moore, J. A. Neuman, J. B. Nowak, B. B. Palm, J. Peischl, F. Piel, P. S. Rickly, A. W. Rollins, T. B. Ryerson, R. H. Schwantes, K. Sekimoto, L. Thornhill, J. A. Thornton, G. S. Tyndall, K. Ullmann, P. Van Rooy, P. R. Veres, C. Warneke, R. A. Washenfelder, A. J. Weinheimer, E. Wiggins, E. Winstead, A. Wisthaler, C. Womack, S. S. Brown, Nighttime and daytime dark oxidation chemistry in wildfire plumes: An observation and model analysis of FIREX-AQ aircraft data. *Atmos. Chem. Phys.* **21**, 16293–16317 (2021).

66. A. K. Mebust, R. C. Cohen, Space-based observations of fire NO*x* emission coefficients: A global biome-scale comparison. *Atmos. Chem. Phys.* **14**, 2509–2524 (2014).

67. X. Jin, Q. Zhu, R. C. Cohen, Direct estimates of biomass burning NO*x* emissions and lifetimes using daily observations from TROPOMI. *Atmos Chem Phys* **21**, 15569–15587 (2021).

68. P. S. Rickly, M. M. Coggon, K. C. Aikin, I. Raul, J. Alvarez, S. Baidar, J. B. Gilman, G. I. Gkatzelis, C. Harkins, J. He, A. Lamplugh, A. O. Langford, B. C. McDonald, J. Peischl, M. A. Robinson, A. W. Rollins, R. H. Schwantes, C. J. Senff, C. Warneke, S. S. Brown, Influence of wildfire on urban ozone: An observationally constrained box modeling study at a site in the Colorado Front Range. *Environ. Sci. Technol.* **57**, 1257–1267 (2023).

69. K. M. Emmerson, M. J. Evans, Comparison of tropospheric gas-phase chemistry schemes for use within global models. *Atmos. Chem. Phys.* **9**, 1831–1845 (2009).

70. S. Myriokefalitakis, N. Daskalakis, A. Gkouvousis, A. Hilboll, T. Van Noije, J. E. Williams, P. Le Sager, V. Huijnen, S. Houweling, T. Bergman, J. Rasmus Nüß, M. Vrekoussis, M. Kanakidou, M. C. Krol, Description and evaluation of a detailed gas-phase chemistry scheme in the TM5-MP global chemistry transport model (r112). *Geosci. Model Dev.* **13**, 5507–5548 (2020).

71. W. Permar, C. Wielgasz, L. Jin, X. Chen, M. Coggon, L. A. Garofalo, G. Gkatzelis, D. Ketcherside, D. B. Millet, B. B. Palm, Q. Peng, M. A. Robinson, J. A. Thornton, P. R. Veres, C. Warneke, R. J. Yokelson, E. V. Fischer, L. Hu, Assessing formic and acetic acid emissions and chemistry in western U.S. wildfire smoke: Implications for atmospheric modeling. *Environ. Sci.: Atmos.* **3**, 1620 (2023).

72. W. S. Goliff, W. R. Stockwell, C. V. Lawson, The regional atmospheric chemistry mechanism, version 2. *Atmos. Environ.* **68**, 174–185 (2013).

73. E. V. Fischer, D. J. Jacob, R. M. Yantosca, M. P. Sulprizio, D. B. Millet, J. Mao, F. Paulot, H. B. Singh, A. Roiger, L. Ries, R. W. Talbot, K. Dzepina, S. Pandey Deolal, Atmospheric peroxyacetyl nitrate (PAN): A global budget and source attribution. *Atmos.Chem. Phys.* **14**, 2679–2698 (2014).

74. H. B. Singh, L. J. Salas, R. B. Chatfield, E. Czech, A. Fried, J. Walega, M. J. Evans, B. D. Field, D. J. Jacob, D. Blake, B. Heikes, R. Talbot, G. Sachse, J. H. Crawford, M. A. Avery, S. Sandholm, H. Fuelberg, Analysis of the atmospheric distribution, sources, and sinks of oxygenated volatile organic chemicals based on measurements over the Pacific during TRACE-P. *J. Geophys. Res., D: Atmos.* **109**, doi.org/10.1029/2003JD003883 (2004).

75. A. W. Rollins, A. Kiendler-Scharr, J. L. Fry, T. Brauers, S. S. Brown, H. P. Dorn, W. P. Dubé, H. Fuchs, A. Mensah, T. F. Mentel, F. Rohrer, R. Tillmann, R. Wegener, P. J. Wooldridge, R. C. Cohen, Isoprene oxidation by nitrate radical: Alkyl nitrate and secondary organic aerosol yields. *Atmos. Chem. Phys.* **9**, 6685–6703 (2009).

76. A. M. Yáñez-Serrano, A. C. Nölscher, E. Bourtsoukidis, B. Derstroff, N. Zannoni, V. Gros, M. Lanza, J. Brito, S. M. Noe, E. House, C. N. Hewitt, B. Langford, E. Nemitz, T. Behrendt, J. Williams, P. Artaxo, M. O. Andreae, J. Kesselmeier, Atmospheric mixing ratios of methyl ethyl ketone (2-butanone) in tropical, boreal, temperate and marine environments. *Atmos. Chem. Phys.* **16**, 10965–10984 (2016).

77. J. R. Schroeder, J. H. Crawford, A. Fried, J. Walega, A. Weinheimer, A. Wisthaler, M. Müller, T. Mikoviny, G. Chen, M. Shook, D. R. Blake, G. S. Tonnesen, New insights into the column CH_2O/NO_2 ratio as an indicator of near-surface ozone sensitivity. *J. Geophys. Res. Atmos.* **122**, 8885–8907 (2017).

78. M. Müller, T. Mikoviny, S. Feil, S. Haidacher, G. Hanel, E. Hartungen, A. Jordan, L. Märk, P. Mutschlechner, R. Schottkowsky, P. Sulzer, J. H. Crawford, A. Wisthaler, A compact PTR-ToF-MS instrument for airborne measurements of volatile organic compounds at high spatiotemporal resolution. *Atmos. Meas. Tech.* **7**, 3763–3772 (2014).

79. G. W. Sachse, G. F. Hill, L. O. Wade, M. G. Perry, Fast-response, high-precision carbon monoxide sensor using a tunable diode laser absorption technique. *J. Geophys. Res. Atmos.* **92**, 2071–2081 (1987).

80. V. Selimovic, R. J. Yokelson, C. Warneke, J. M. Roberts, J. De Gouw, J. Reardon, D. W. T. Griffith, Aerosol optical properties and trace gas emissions by PAX and OP-FTIR for

laboratory-simulated western US wildfires during FIREX. *Atmos. Chem. Phys.* **18**, 2929–2948 (2018).

81. B. A. Ridley, F. E. Grahek, J. G. Walega, A small high-sensitivity, medium-response ozone detector suitable for measurements from light aircraft. *J. Atmos. Oceanic Tech.* **9**, 142–148 (1992).

82. R. E. Shetter, W. Junkermann, W. H. Swartz, G. J. Frost, J. H. Crawford, B. L. Lefer, J. D. Barrick, S. R. Hall, A. Hofzumahaus, A. Bais, J. G. Calvert, C. A. Cantrell, S. Madronich, M. Müller, A. Kraus, P. S. Monks, G. D. Edwards, R. McKenzie, P. Johnston, R. Schmitt, E. Griffioen, M. Krol, A. Kylling, R. R. Dickerson, S. A. Lloyd, T. Martin, B. Gardiner, B. Mayer, G. Pfister, E. P. Röth, P. Koepke, A. Ruggaber, H. Schwander, M. van Weele, Photolysis frequency of NO_2: Measurement and modeling during the International Photolysis Frequency Measurement and Modeling Intercomparison (IPMMI). *J. Geophys. Res. Atmos.* **108**, 8544 (2003).

83. B. Lebegue, M. Schmidt, M. Ramonet, B. Wastine, C. Yver Kwok, O. Laurent, S. Belviso, A. Guemri, C. Philippon, J. Smith, S. Conil, Comparison of nitrous oxide (N_2O) analyzers for high-precision measurements of atmospheric mole fractions. *Atmos. Meas. Tech.* **9**, 1221–1238 (2016).

84. B. H. Lee, F. D. Lopez-Hilfiker, P. R. Veres, E. E. McDuffie, D. L. Fibiger, T. L. Sparks, C. J. Ebben, J. R. Green, J. C. Schroder, P. Campuzano-Jost, S. Iyer, E. L. D'Ambro, S. Schobesberger, S. S. Brown, P. J. Wooldridge, R. C. Cohen, M. N. Fiddler, S. Bililign, J. L. Jimenez, T. Kurtén, A. J. Weinheimer, L. Jaegle, J. A. Thornton, Flight deployment of a high-resolution time-of-flight chemical ionization mass spectrometer: Observations of reactive halogen and nitrogen oxide species. *J. Geophys. Res. Atmos.* **123**, 7670–7686 (2018).

85. B. B. Palm, X. Liu, J. L. Jimenez, J. A. Thornton, Performance of a new coaxial ion-molecule reaction region for low-pressure chemical ionization mass spectrometry with reduced instrument wall interactions. *Atmos. Meas. Tech.* **12**, 5829–5844 (2019).

86. W. Zheng, F. M. Flocke, G. S. Tyndall, A. Swanson, J. J. Orlando, J. M. Roberts, L. G. Huey, D. J. Tanner, Characterization of a thermal decomposition chemical ionization mass

spectrometer for the measurement of peroxy acyl nitrates (PANs) in the atmosphere. *Atmos. Chem. Phys.* **11**, 6529–6547 (2011).

87. S. R. Hall, K. Ullmann, M. J. Prather, C. M. Flynn, L. T. Murray, A. M. Fiore, G. Correa, S. A. Strode, S. D. Steenrod, J. F. Lamarque, J. Guth, B. Josse, J. Flemming, V. Huijnen, N. Luke Abraham, A. T. Archibald, Cloud impacts on photochemistry: Building a climatology of photolysis rates from the Atmospheric Tomography mission. *Atmos. Chem. Phys.* **18**, 16809–16828 (2018).

88. P. R. Veres, J. M. Roberts, R. J. Wild, P. M. Edwards, S. S. Brown, T. S. Bates, P. K. Quinn, J. E. Johnson, R. J. Zamora, J. De Gouw, Peroxynitric acid (HO_2NO_2) measurements during the UBWOS 2013 and 2014 studies using iodide ion chemical ionization mass spectrometry. *Atmos. Chem. Phys.* **15**, 8101–8114 (2015).

89. I. Bourgeois, J. Peischl, C. R. Thompson, K. C. Aikin, T. Campos, H. Clark, R. Commane, B. Daube, G. W. Diskin, J. W. Elkins, R. S. Gao, A. Gaudel, E. J. Hintsa, B. J. Johnson, R. Kivi, K. McKain, F. L. Moore, D. D. Parrish, R. Querel, E. Ray, R. Sánchez, C. Sweeney, D. W. Tarasick, A. M. Thompson, V. Thouret, J. C. Witte, S. C. Wofsy, T. B. Ryerson, Global-scale distribution of ozone in the remote troposphere from the ATom and HIPPO airborne field missions. *Atmos. Chem. Phys.* **20**, 10611–10635 (2020).
